# Respiratory allergic diseases and allergen immunotherapy: A French patient survey before and during the COVID-19 pandemic^☆^

**DOI:** 10.1016/j.waojou.2024.100902

**Published:** 2024-04-10

**Authors:** Philippe Devillier, Sarah Saf, Christine Rolland, Giorgio Walter Canonica, Pascal Demoly

**Affiliations:** a VIM (Laboratoire de Recherche en Pharmacologie respiratoire, Virologie et Immunologie Moléculaire) Suresnes – UMR_0892 & Clinical Research Unit, Foch Hospital, University Versailles Saint-Quentin, Suresnes, France; b Department of Pediatric, Hospital Centre of Arpajon, Arpajon, France; c Department of Pediatric Allergology, Armand Trousseau University Hospital, Paris, France; d Asthma & Allergies Association of Patients, Paris, France; e Department of Biomedical Sciences, Humanitas University, Milan, Italy; f Personalized Medicine Asthma & Allergy Unit-IRCCS, Humanitas Research Hospital, Milan, Italy; g University Hospital of Montpellier and IDESP, University of Montpellier – INSERM – INRIA PreMediCal, Montpellier, France

**Keywords:** COVID-19, Allergen immunotherapy, Respiratory allergy, Allergic rhinitis, Asthma

## Abstract

**Background:**

The COVID-19 pandemic brought unprecedented global disruption to both healthcare providers and patients with respiratory allergies. There are limited real-life data on the impact of the COVID-19 pandemic on the risk perception of patients with allergy treated with allergen immunotherapy (AIT).

**Objective:**

To understand the risk perception of allergic patients treated with sublingual immunotherapy (SLIT) before and during the pandemic, and their attitudes towards COVID-19 infection and vaccination.

**Methods:**

This was a non-interventional, cross-sectional survey conducted from October to November 2021 in France. Adult patients, who had been prescribed and had received a Stallergenes SLIT (liquid or liquid and tablets) before the pandemic (from August 1, 2018 to March 10, 2020) and during the pandemic (from March 11, 2020 to August 31, 2021), were identified from the Stallergenes named-patient products (NPP) database. Patients completed an online questionnaire. Data were analyzed descriptively.

**Results:**

A total of 5258 patients from all over France completed the questionnaire. Mean (±SD) age of the respondents was 39.3 (±13.0) years and 66.9% were female. Some of them (11.8%) were obese (BMI >30 kg/m^2^). Main allergic diseases were rhinitis (80.0% of patients) with or without conjunctivitis, and asthma (39.0%). More than half of the patients experienced moderate to severe (58.0%) and persistent allergic rhinitis profile (70.4%). Most patients were poly-allergic (72.7%), mostly to house dust mites (61.9%), grass pollens (61.5%), tree pollens (57.8%), and cat dander (37.2%). Only 14.1% of patients experienced an aggravation of their allergy symptoms during lockdown and 14.8% were infected with COVID-19, with hospitalization required for 1.8%. Only 3.1% of patients reported their SLIT initiation as being postponed due to the pandemic. SLIT was changed, temporarily interrupted or permanently discontinued during the pandemic in 21.9% of patients. Changes mainly concerned the maintenance dose for SLIT-liquid (63.2%). SLIT modification was due to COVID-19 infection in only 4.2%. Most patients did not feel vulnerable (53.1%), anxious (55.2%), at risk to present severe symptoms of COVID-19 (77.1%), or at risk to transmit coronavirus (80.4%). However, greater anxiety was reported in patients with allergic asthma (33.6%) or other respiratory disorders (50.4%). Patients who felt vulnerable partly assigned their vulnerability to their allergic disease (59.3%). Suffering from an allergic disease did not make patients feel more vulnerable to side effects of COVID-19 vaccine for 79.6% of them.

**Conclusion:**

Overall, most patients with allergy and under SLIT were not strongly concerned by the COVID-19 infection. SLIT did not have a negative impact on the COVID-19 symptoms.

## Introduction

The COVID-19 disease pandemic caused by the respiratory syndrome coronavirus-2 (SARS-CoV-2) brought unprecedented disruption and challenges to healthcare throughout the world.^1^, ^2^, ^3^ Clinicians providing care for patients with chronic allergies had to continuously adjust their medical practice to restrictions imposed by authorities.^4^, ^5^, ^6^, ^7^ Likewise, patients with respiratory allergies faced uncertainty regarding the potential severity of an infection with the virus, or its effects on their ongoing therapies, and this increased their anxiety and restraint in daily activities.^8^, ^9^, ^10^ As some allergic diseases may present symptoms which are in differential diagnosis with COVID-19, such as coughing and sneezing, guidance was offered to differentiate the diseases.^11^^,^^12^ Similarly, recommendations for improved management of allergic respiratory diseases were issued by allergy societies in several countries.^13^, ^14^, ^15^, ^16^, ^17^ Guidance was also given for continuation of treatment with allergen immunotherapy (AIT) to control allergic diseases and surveillance of patients on these treatments during the pandemic.^16^^,^^18^^,^^19^

France was among the 10 countries most severely affected by the COVID-19 pandemic worldwide. According to the World Health Organization (WHO), there were 39,068,474 confirmed cases of COVID-19 with 163,787 deaths in France alone from January 2020 to June 2023.^20^ Real-life data on the impact of COVID-19 pandemic on the health of patients with allergy treated with AIT in France remain limited.^10^ For example, the pandemic could have affected attitudes towards AIT and adherence to treatment, willingness to COVID-19 vaccination, or altered levels of anxiety due to the allergy. For this reason, Stallergenes decided to conduct a non-interventional, cross-sectional public interest survey of sublingual immunotherapy (SLIT)-treated patients with allergy using their named-patient products (NPP) database in France. The main objective was to understand the risk perception of patients with allergy treated with SLIT and their attitudes towards COVID-19 infection and vaccination before and during the COVID-19 pandemic.

## Materials and methods

This was a non-interventional, cross-sectional survey conducted from the October 21, 2021 to the November 19, 2021 in France. The survey required the completion of an e-questionnaire by patients with respiratory allergic diseases and treated with SLIT before and during the pandemic. Following the French Data Protection law, no submission of the study protocol to the opinion of an Ethics Committee or authorization from the French Health Authority (ANSM) was required. The protocol collected data and results were submitted to Health Data Hub, the French public registry of health data.

### Participants

Patients included in the survey were those aged 18 years old or more and in the Stallergenes-NPP database, who had been prescribed a Stallergenes SLIT (liquid or liquid and tablets) from August 1, 2018 to August 31, 2021. In addition, patients had to have received at least 1 Stallergenes NPP from August 1, 2018 to March 10, 2020 (before the pandemic) and from March 11, 2020 to August 31, 2021 (during the pandemic). Patients who provided a mobile phone number for delivery of their treatment at home, and who agreed to be contacted, were invited to participate in the survey. Those who did not have SLIT treatment initiated before the pandemic and whose treatment postponement was not due to COVID-19 were removed from the analysis population as they were not concerned by the survey. Physicians (N = 3684) who had prescribed at least 1 SLIT using Stallergenes-NPP from August 1, 2018 to August 31, 2021, recorded in the Veeva database, were made aware of the survey to be able to answer, if needed, to patients seeking further information, but did not participate as this survey was designed to be completed only by patients.

### Survey procedures

The invitation for survey participation was sent by text message from a third-party provider having access only to patient's mobile phone numbers and no other patient's information. The invitation contained a brief message as well as a URL link. By clicking on this URL link, patients were routed to the information note they could read and download to keep a copy. Patients who agreed to participate validated their consent and had access, via a second link, to the actual e-questionnaire for completion.

The e-questionnaire was composed of 17 questions in 6 major modules: patient profile, patient allergic disease, COVID-19 infection, treatment with AIT or desensitization, COVID-19 risk perception, and COVID-19 vaccination (See Appendix A). Each module included several tabs. Patients were asked to complete it at once. Once a tab including several questions was saved, patients could not return to change their response. Follow-up reminders were sent by text message to all participating patients 3 times during the survey period, to ensure maximal response rate.

### Statistical analysis

Since this was a survey, no formal calculation of sample size was performed. Assuming a response rate of 3–5% of patients participating and completing (at least partially) the e-questionnaire, the expected sample size was 3700 to 6100 patients fulfilling the predefined eligibility criteria in the Stallergenes-NPP database. Data were analyzed descriptively. Summary measures of all continuous and ordinal variables included the number of patient (n), mean and standard deviation. Summary measures for all discrete variables included the frequency counts and percentage by category. All computation and generation of tables were performed using Statistical Analysis Systems (SAS®, SAS Institute Inc., Cary, NC, USA) release 9.4.

## Results

From the 195,000 individuals in the Stallergenes-NPP database who received at least 1 SLIT between August 1, 2018 to August 31, 2021, 122,084 adult patients gave their consent to be contacted and were invited to participate in the survey. Of these, 5258 patients (4.3%) completed the e-questionnaire and 3723 (70.8%) were included in the final analysis (Appendix B, Fig. S1). A total of 1532 patients were excluded from the analysis as they did not initiate their desensitization treatment before the pandemic and the treatment postponement was not due to COVID-19 pandemic. The analysis of this sub-population showed no difference from the population in the main analysis.

### Patient profile and allergic disease

The mean (±SD) age of respondents was 39.3 (±13.0) years with a vast majority (78.9%) under 49 years, and 66.9% were female (Table 1). Some of them (24.5%) were overweight (BMI = 25–30 kg/m^2^) and 11.8% were obese (BMI >30 kg/m^2^). All 13 French regions were represented with the largest number of respondents living in Ile-de-France (21.7%), Auvergne-Rhône-Alpes (12.7%), Occitanie (10.3%) and Provence-Alpes-Côte d'Azur (9.8%), reflecting the regions most affected by the pandemic in 2020 and 2021 (Appendix B, Table S1). Allergic rhinitis (AR) with or without conjunctivitis and allergic asthma were the main allergic diseases, affecting 80.0% and 39.0% of the patients surveyed, respectively. The most common allergens responsible were house dust mites (HDM, 61.9% of patients), grass pollens (61.5%), and tree pollens (57.8%). Most patients (72.7%) declared being poly-allergic. According to Allergic Rhinitis and its Impact on Asthma (ARIA) classification of AR, 47.1% of the patients suffered from persistent moderate to severe AR and 23.3% from persistent mild AR. More than a half of patients (52.8%) had been suffering from allergic respiratory disease for 15 years or more. Very few patients (8.5%) presented risk criteria for severe COVID-19 infection (defined as reporting at least 1 comorbidity). Among these patients, the main comorbidities were metabolism and nutrition disorders (59.9%), other respiratory, thoracic and mediastinal disorders (36.3%, eg, bronchitis, cystic fibrosis, sleep apnoea), and cardiac disorders (24.0%).

**Table 1:** Demographic and clinical characteristics of the patients.

Variable	N = 3723
Age *(n=3632)*, years, mean ± SD	39.3 ± 13.0
Gender *(n=3718)*, female	2487 (66.9)
BMI *(n=3649)*, kg/m^2^, mean ± SD	24.4 ± 4.6
<25 kg/m^2^	2315 (63.7)
25–30 kg/m^2^	894 (24.5)
>30 kg/m^2^	430 (11.8)
Allergic disease	
Allergic rhinitis with or without conjunctivitis	2980 (80.0)
Allergic asthma	1451 (39.0)
Food allergy	543 (14.6)
Atopic dermatitis	351 (9.4)
Confirmed drug allergy	168 (4.5)
Hymenoptera venom allergy^a^	123 (3.3)
Allergic status *(n=3716)*	
Mono-allergic	1015 (27.3)
Poly-allergic	2701 (72.7)
Allergen families^b^	
HDM	2306 (61.9)
Grass pollens	2288 (61.5)
Tree pollens	2153 (57.8)
Cat dander	1386 (37.2)
Weed pollens	840 (22.6)
Mould	632 (17.0)
Severity of AR^c^*(n=2965)*	
Mild	1244 (42.0)
Moderate to severe	1721 (58.0)
Symptoms frequency^d^*(n=2943)*	
Intermittent (≤4 weeks per year)	871 (29.6)
Persistent (>4 weeks per year)	2072 (70.4)
ARIA classification of AR (frequency∗severity) *(n=2932)*	
Intermittent mild	541 (18.5)
Persistent mild	648 (23.3)
Intermittent moderate to severe	327 (11.2)
Persistent moderate to severe	1380 (47.1)
Duration of allergic respiratory disease *(n=3677)*	
≤1–4 years	403 (11.0)
5–14 years	1332 (36.2)
≥15 years	1942 (52.8)
Presented severe COVID-19 infection risk^e^*(n=3715)*	317 (8.5)
SOC classification of comorbidities *(n=302)*	
Metabolism and nutrition disorders	190 (59.9)
Respiratory, thoracic and mediastinal disorders	115 (36.3)
Cardiac disorders	76 (24.0)
Neoplasms benign, malignant and unspecified	15 (4.7)
Other	4 (1.2)

All values represent n (%) unless otherwise indicated. N, number of patients in the main analysis; n, number of patients with data; SOC, system organ class.

a Wasp, bee, hornet, and bumblebee.

b Grass pollens (cocksfoot, timothy, sweet vernal grass, meadow grass and rye grass); tree pollens (alder, birch, ash, hazel, olive, Alternaria), weed pollens (ragweed, mugwort, pellitory); mould (Alternaria).

c Mild: normal sleep, normal social and leisure activities, normal school or work activities and symptoms present but not troublesome; Moderate to severe (with at least 1 sign): disturbed sleep, disturbed social and leisure activities, disturbed school or work activities, troublesome symptoms.

d ARIA classification of allergic rhinitis (revised 2010).

e List of risk factors for severe forms of COVID-19: history of cardiovascular disease (heart failure, hypertension, chest pain), diabetes, obesity, chronic respiratory diseases (bronchitis, cystic fibrosis, sleep apnoea), cancer, renal failure, cirrhosis, splenectomy, sickle cell anemia

### COVID-19 infection

Issues directly related to the COVID-19 pandemic affecting patients under SLIT are shown in Table 2. Few patients (14.1%) experienced an aggravation of their allergy symptoms during lockdown and almost 60.0% of the patients were able to consult a physician as much as desired during this period. Patients were generally not bothered because of their allergy when wearing the mask (57.2%), except for those reporting respiratory disorders and those who initiated their treatment after the beginning of the pandemic (67.0% and 68.9%, respectively, reported some discomfort). Only 22.2% of the patients reported having mistaken their allergic symptoms with COVID-19 ones. Some respondents (14.8%) reported they had been tested positive for COVID-19 but, of these, only a very small proportion (1.8%) required hospitalization. In our survey, a focus on these infected hospitalized patients compared to non-hospitalized patients and non-infected ones showed the former had more atopic dermatitis and Hymenoptera venom allergic diseases, presented more persistent moderate to severe AR, were mainly allergic to tree pollens, often had an AR ≥15 years and their main comorbidities were cardiac disorders and metabolism/nutrition disorders (Appendix B, Table S2). No notable differences were observed in patients with or without allergic asthma with regards to issues related to the pandemic. However, asthmatic patients reported more confusion between their allergy and COVID-19 symptoms than non-asthmatic patients (28.0% versus 18.6%). Markedly, more asthmatic patients felt discomfort of wearing the mask (51.6%) as opposed to non-asthmatic patients (37.2%). Only a small percentage of asthmatic and non-asthmatic patients were infected with COVID-19 (15.8% and 14.1%, respectively).

**Table 2:** COVID-19 infection in the analysis population.

	N = 3723
Confused allergy and COVID-19 symptoms^a^*(n=3699)*	823 (22.2)
Allergy symptoms aggravated during lockdown *(n=3698)*	520 (14.1)
Consulted physician during lockdown as much as desired *(n=3682)*	2156 (58.6)
Type of consultation *(n=2129)*	
Physical	1237 (58.1)
Remote	605 (28.5)
Physical and remote	285 (13.4)
Felt discomfort of wearing a mask *(n=3700)*	1583 (42.8)
Infected with COVID-19 *(n=3706)*	547 (14.8)
Type of COVID-19 diagnosis *(n=545)*	
Confirmed by a doctor	44 (8.1)
Diagnosed by a positive PCR or antigen test	350 (64.2)
Diagnosed by a test and confirmed by a doctor	151 (27.7)
Presented COVID-19 symptoms *(n=547)*	479 (87.6)
COVID-19-related care *(n=556)*	
Simple isolation	386 (69.4)
Home isolation with medication	160 (28.8)
Hospitalization	10 (1.8)
Fully recovered from COVID-19 *(n=546)*	448 (82.1)
Duration of symptoms in those not fully recovered *(n=98)*	
<15 days (recent or ongoing infection)	4 (4.1)
Between 15 days and 12 weeks	9 (9.2)
>12 weeks (long COVID)	85 (86.7)

All values are n (%). N, number of patients in the main analysis; n, number of patients with data.

a COVID-19 symptoms: cough, sneezing, headaches, loss of smell or taste, fatigue

In general, most patients recovered entirely (82.1%); for those who did not recover, 86.7% reported suffering for long-term symptoms for longer than 12 weeks (long COVID). A focus on these long-COVID-suffering patients compared to those with short duration of COVID-19 symptoms and non-infected patients showed the former had more atopic dermatitis and food allergy, presented more persistent moderate to severe AR, were poly-allergic with mainly tree pollen allergies, presented risk criteria for severe COVID-19 infection and their main comorbidities were metabolism and nutrition disorders (Appendix B, Table S3).

### Allergen immunotherapy

The survey results relative to SLIT are shown in Table 3. Most patients (78.8%) were prescribed their SLIT in liquid form and 21.2% were prescribed their SLIT in both liquid and tablet forms, associated to a corticosteroid-based treatment for 35.9% of them. Several patients reported multiple combinations of corticosteroids associating systemic and topical routes (Table 3). The systemic route was defined as corticosteroid treatment administered by oral route and/or injection with or without topical corticosteroids, and concerned 4.4% of patients. An analysis of the population taking corticosteroids compared to those with no corticosteroid-based treatment showed the former had more allergic asthma, atopic dermatitis and food allergy, presented more persistent moderate to severe AR, were poly-allergic with mainly HDM allergy, often had an AR ≥15 years, had very few risk criteria for severe COVID-19 infection (13.9% of obese patients, with 16.6% taking systemic CS, versus 10.7%) but no more comorbidities except for respiratory, thoracic and mediastinal disorders (Appendix B, Table S4).

**Table 3:** Allergen immunotherapy.

	N = 3723
Type of treatment *(n=3704)*	
Liquid	2919 (78.8)
Liquid and tablet	785 (21.2)
Initiated SLIT before pandemic *(n=3707)*	3593 (96.9)
If SLIT initiated before pandemic, modification of treatment during pandemic *(n=3586)*	
Unchanged	2803 (78.2)
Changed	68 (1.9)
Temporary interruption	235 (6.6)
Permanently discontinued	480 (13.4)
If treatment changed, type of modification *(n=68)*	
Initiation dose	18 (26.5)
Maintenance dose	43 (63.2)
Duration of treatment	7 (10.3)
If treatment changed, temporary interrupted, or permanently discontinued, allergic symptoms reappeared *(n=781)*	491 (62.9)
If treatment changed, temporary interrupted or permanently discontinued, caused by COVID-19 infection or positive diagnosis *(N=783)*	33 (4.2)
SLIT not initiated before pandemic *(n=91)*	
Treatment postponed because of pandemic	91 (100)
Patients with corticosteroid-based treatment *(n=3687)*	1325 (35.9)^a^
Ocular route	160 (4.3)
Nasal route	570 (15.5)
Inhalation	1031 (28.0)
Cutaneous route	80 (2.2)
Oral route or injection (systemic)	162 (4.4)

All values are n (%). N, number of patients in the main analysis; n, number of patients with data.

SLIT, sublingual immunotherapy.

Non-exhaustive list of corticosteroid-based treatment in the context of allergies: beclomethasone, budesonide, dexamethasone, fluticasone, mometasone, prednisolone, tixocortol, triamcinolone

a % patients taking CS by the different routes.

Only 3.1% of respondents reported their SLIT initiation as being postponed due to the pandemic. SLIT was changed, temporary interrupted or permanently discontinued during the pandemic in 21.9% of patients, rarely due to COVID-19 infection (4.2%). For those who initiated SLIT before the pandemic, changes mainly concerned the maintenance dose for SLIT-liquid (63.2%) versus the initiation dose (26.5%) or the duration of treatment (10.3%). SLIT-tablets being non-breakable, the maintenance dose is constant and was not expected to be changed. Treatment interruption or discontinuation led to symptoms reappearance in 62.9% of the patients.

### COVID-19 risk perception

**Fig. 1:**
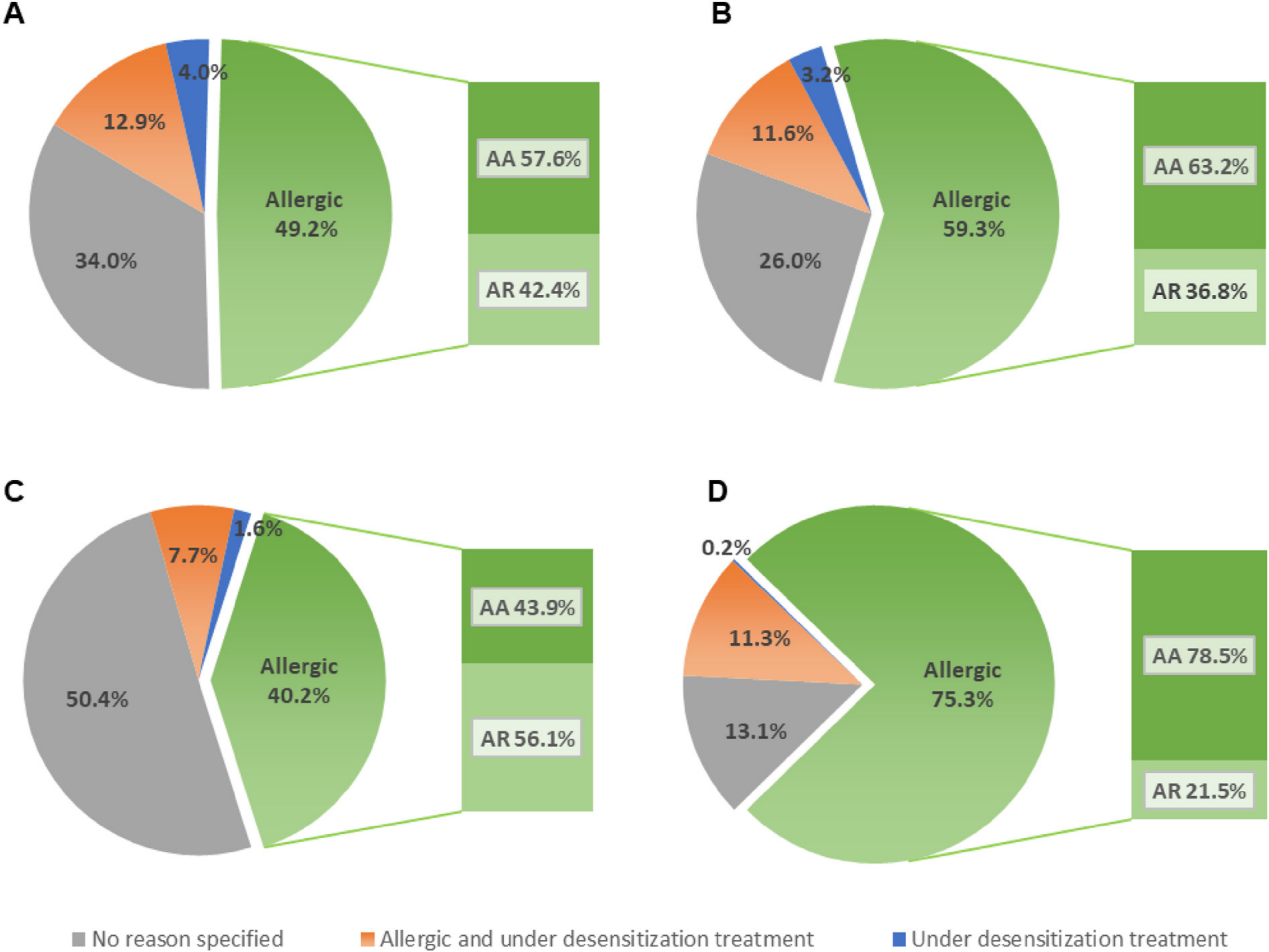
Reasons for feeling A) anxiety (n = 1664), B) vulnerability (n = 1740), C) at risk to transmit the coronavirus (n = 728), D) at risk to present severe symptoms of coronavirus (n = 846)

The majority of the patients did not feel vulnerable (53.1%), anxious (55.2%), at risk to present severe symptoms of COVID-19 (77.1%), or at risk to transmit coronavirus (80.4%) (Fig. 1 and Table S5). For the patients who felt anxious, being allergic was the main reason for 49.2% of them. And for those patients, asthma was the main allergic disease (57.6%). Similarly, 59.3% patients who felt vulnerable assigned their vulnerability to their allergic disease, of whom 63.2% had allergic asthma, and 75.3% of the patients who felt at risk to present severe symptoms of coronavirus were also suffering from allergic asthma (78.5%). In contrast, 40.2% of the allergic patients who felt at risk to transmit the coronavirus were suffering mainly from allergic rhinoconjunctivitis (56.1%) instead of allergic asthma. Of note, a focus on the population who ticked obesity as comorbidity showed they felt very/extremely vulnerable, and at greater risk to transmit the coronavirus and to present severe symptoms of coronavirus than non-obese patients (Appendix B, Table S6).

### COVID-19 vaccination

**Fig. 2:**
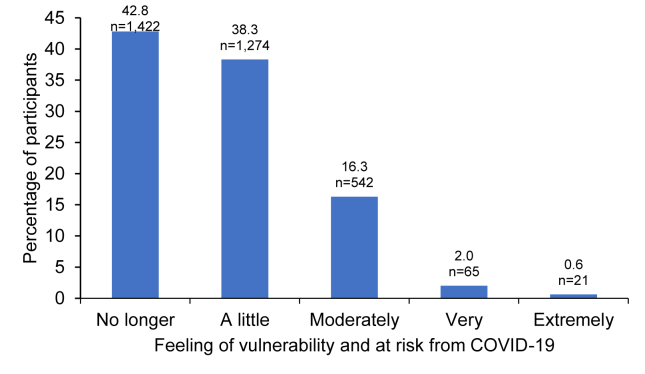
Feeling of vulnerability and at risk from COVID-19 in patients who received the COVID-19 vaccine (n = 3324)

A total of 3335 (90.0%) of the patients reported having received the COVID-19 vaccine. Since vaccination, 81.1% of the patients felt no longer or just a little at risk from COVID-19 (Fig. 2). Suffering from an allergic disease did not make patients feeling more vulnerable to side effects of COVID-19 vaccine for 79.6% of them.

### Subgroup analyses

The effects on SLIT treatment and on the perception towards COVID-19 were further analyzed by subgroups according to age, type of comorbidities, duration of allergic respiratory disease, allergic profile, and asthma status (Appendix B, Tables S7-S15).

Most of the patients who permanently discontinued SLIT during the pandemic were those aged below 49 years (>12.0% versus <12.0% in older patients). The 2 extreme age classes were less frequently (<30.0%) under corticosteroid-based treatment as compared to the patients aged 30 up to 74 (≈38.0%). All age classes mostly did not feel anxious (52.4%–61.2%) or vulnerable at all (40.9%–61.4%) (Table S7-S8).

Patients with cardiac disorders were not prescribed mixed SLIT treatment (liquid and tablet forms) (11.8%) as often as other groups (17.6%–23.7%) and corticosteroid-based treatment was more often given to patients with respiratory, thoracic and mediastinal disorders (63.2%) than other groups (Table S9). Patients with respiratory, thoracic and mediastinal disorders reported feeling at least a little anxiety (71.3%)/vulnerability (80.7%) more frequently than any other group (67.2%/72.1% for patients with cardiac disorders; 63.8%/70.6% for patients with metabolism and nutrition disorders and 66.7%/53.3% for patients with neoplasms benign/malignancy). This feeling of anxiety/vulnerability was mainly driven by their allergy (70.7%/69.6%); more specifically 84.5%/87.5% of allergic asthma versus 15.5%/12.5% of allergic rhinoconjunctivitis (Table S10).

Regarding duration of allergic respiratory disease, results were very similar between groups for SLIT treatment (Table S11). Only 28.0% of patients that were allergic for 4 years or less received corticosteroids compared to 31.5% of patients allergic for [5-14] years and 40.9% of the patients allergic ≥15 years. A smaller proportion of patients allergic for ≥15 years were feeling not at all anxious/vulnerable compared to the 2 other categories (51.6%/49.2% versus 59.0%–60.0%/56.7%–57.5%) for the other groups] (Table S12).

Poly-allergic patients reported a higher rate of combined SLIT treatment (liquid and tablet forms) than mono-allergic patients (23.8% versus 14.3%) (Table S13). Modifications of treatment were markedly different among patients: more poly-allergic patients had a modification of their initiation dose than mono-allergic patients (30.4% versus 8.3%); less poly-allergic patients had modification of maintenance of their dose or modification of the duration of their treatment than mono-allergic patients (60.7% and 8.9% versus 75.0% and 16.7%, respectively). Mono-allergic patients were less frequently (27.9%) under corticosteroid-based treatment as compared to poly-allergic ones (39.0%). Less poly-allergic patients did not feel anxious (52.4% versus 62.8%) or vulnerable (50.2% versus 60.8%) at all than mono-allergic ones (Table S14).

COVID-19 risk perception according to asthmatic status showed there were more non-asthmatic patients who reported that they did not feel any anxiety/vulnerability at all as compared to asthmatic ones (60.7% versus 46.7%/62.7% versus 38.1%, respectively) (Table S15).

## Discussion

The objective of the Stallergenes “Respiratory Allergies and COVID-19” (cross-sectional survey was to evaluate the impact of the COVID-19 pandemic in patients with allergy treated with SLIT in France. Generally, the survey showed that most patients with allergy and under SLIT were not strongly concerned by a potential infection with COVID-19. Further, SLIT did not have a negative impact on the COVID-19 symptoms.

AR with or without conjunctivitis and allergic asthma were the main allergic diseases affecting the patients in our survey. Additionally, 72.7% of the patients declared they were poly-allergic, a rate consistent with a previous survey conducted in France on polysensitization to aeroallergens, in which 70.2% of the polysensitized patients were affected.^21^ Similar findings were also observed regarding the main allergens that were HDM (61.9% v. 79.8%), grass pollens (61.5% v. 74.1%), tree pollens (57.8% v. 59.5%), cat dander (37.2% v. 42.1%), weed pollens (22.6% v. 24.6%), and mould (17.0% v. 16.2%) for our survey and the published report.^21^ More than half of the patients experienced moderate to severe AR and had a persistent AR profile. The most severe form of AR according to ARIA classification, persistent moderate to severe was observed in 47.1% of cases. A duration of allergic respiratory disease for ≥15 years was noted in more than half of the respondents. Very few patients in the survey reported suffering from a comorbidity. Among these patients, the main comorbidities were metabolic and nutrition disorders, respiratory, thoracic and mediastinal disorders, and cardiac disorders. Although some early data suggested higher rate of asthma in patients hospitalized for severe COVID-19 illness,^22^^,^^23^ several recent meta-analyses found that the risk of contracting COVID-19 was actually lower in patients with asthma versus non-asthmatics, and that there were no significant differences in hospitalization, intensive care admission, mechanical ventilation requirements, or death in these patients.^24^^,^^25^ Generally, asthma severity or the use of asthma medication were not independent factors for poor clinical outcomes of COVID-19.^23^

In April 2021, it was already estimated that the proportion of the French adult population infected with COVID-19 since the beginning of the pandemic was 22.7%.^26^ This rate decreased progressively to an incidence rate of 55 per 100,000 inhabitants in October 2021 when our survey started (Santé Publique, data generated on October 27, 2021). This decreasing rate was reflected in our study population, as only 14.8% of patients reported to have been infected with COVID-19, medically confirmed, in November 2021. Of these, only 1.8% of them required hospitalization, but no data was collected in our survey on the reasons for hospitalization (severity, indication or duration). In general, patients recovered entirely from COVID-19 infection but for those who did not recover, a majority suffered for long-term symptoms >12 weeks (long COVID). Some of the allergic diseases may present symptoms (nasal congestion, coughing and sneezing) which are in the COVID-19 differential diagnosis.^11^^,^^19^ In our survey, only 22.2% of the patients reported having mistaken their allergic symptoms with COVID-19 ones. Few patients (14.1%) experienced an aggravation of their allergy symptoms during lockdown, and most (60.0%) were able to consult a physician as much as desired during lockdown, mainly in face-to-face consultations (58.1%). This is quite relevant as healthcare continuity was declared a public health imperative in France.^27^ However, a decline in access to care compared to 2019 was observed in the first lockdown: −51.0% among specialist doctors and −25.0% among general physicians.^27^ Concerning allergy related consultations, a recent survey described that Italian physicians (N = 66) did not consider that the COVID-19 pandemic presented an added risk to patients with allergic asthma or rhinitis receiving AIT.^28^ Although most treatments continued, there were reduced rates of AIT initiations and sublingual AIT was favored over subcutaneous AIT. In a survey of German allergists, more than 70.0% of the respondents stated that they regularly initiated and dosed up AIT in healthy patients without current symptoms indicating an infection with COVID-19.^29^ However, in a worldwide survey, about 80.0% of healthcare professionals (HCPs) indicated being significantly affected in their allergy practice. In this survey a reduction in face-to-face visits was reported by 93.0% of HCPs, and about a quarter completely interrupted diagnostic challenges.^30^ Changes in management of allergic patients and increase in use of telemedicine at a high rate during the COVID-19 pandemic for asthma and rhinitis have also been reported.^31^^,^^32^

SLIT was initiated before the pandemic for almost all patients and treatment modification in slightly more than 1 patient on 5 was rarely caused by COVID-19 infection. Although guidance has been given for continuation of ongoing treatment with SLIT to control allergic diseases during the pandemic,^14^^,^^19^ 20.0% of the patients in our survey have seen their treatment temporarily interrupted or permanently discontinued, which led to the reappearance of the symptoms for 62.9% of them. One of the advantages of SLIT liquid form is the flexibility of the dose. In current practice, dose adaptation appears to be common to enable patients to benefit from treatment, whilst maximizing safety and tolerability, as recently evidenced in the MaDo study in France.^33^ In this study, dose adjustments were noted in 65.8% of patients, and for 95.7% of them during the maintenance phase (up and down to a similar extent). The main reasons were a wish for greater efficacy (for dose increase), occurrence of adverse effects, worsening of symptoms (for dose reductions). In the CORAP study, changes concerned less than 2% of patients and during the maintenance phase for 63.2% of them. The reasons for change may have been similar to those reported in the MaDo study and probably not directly related to COVID since 4.2% of patients with treatment modification stated that it was rarely due to the infection.

In our survey, the majority of the patients did not feel at all anxious, vulnerable, at risk to transmit coronavirus, or at risk to present severe symptoms of COVID-19. The French CoviPrev survey showed that 23.0% of people suffered from anxiety related to COVID-19 pandemic in France.^34^ In our population of patients with allergic diseases, a similar proportion of 26.9% of respondents reported COVID-19-related anxiety. For the patients who did feel at least a little anxious or vulnerable, being allergic was the main reason; for those patients, allergic asthma was the main type of allergy. There seemed to be a trend with the youngest being less anxious or vulnerable with COVID-19 compared to the oldest, contrasting with the CoviPrev survey, in which younger age classes were more anxious than older ones.^34^ Overall, current research has shown that the COVID-19 pandemic had a negative psychological impact on people with chronic allergic diseases.^8^ Patients with AR reported higher anxiety and depression scores than healthy controls. Behavior changes during the pandemic, such as mask wearing, spending less time outdoors, more time spent indoors, and decreased air pollution during lockdown periods, could have affected the symptom scores. González-Díaz et al reported that during the lockdown, the psychological impact in patients with allergic diseases was greater compared to individuals without allergy and individuals with respiratory allergy such as asthma and rhinitis were particularly vulnerable to scoring higher in a depression symptoms scale.^35^ Increased symptoms of anxiety, depression and fear during the COVID-19 pandemic were also found in patients with asthma in a study from the Netherlands.^36^ Similarly, asthma was associated with a decline in mental health during lockdown, with increased levels of anxiety, depression and wellbeing particularly among young adults in a study in the United Kingdom.^9^ In a study from the United States, people with asthma reported higher levels of anxiety, perceived stress, and burnout symptoms compared to controls.^37^ Another French survey conducted by a patient association in 2020 showed that more than 80% of asthma patients felt more at risk than the rest of the population about COVID-19 and the very high anxiety, expressed by 84% of them, motivated them to seek validated information.^38^ In contrast, a study from Wuhan (China) reported that the pandemic had no significant negative impact on the allergic rhinitis patients' psychological status.^39^

Interestingly, in our survey, patients presenting 1 or multiple comorbidities felt much more at risk to present severe symptoms of COVID-19 (46.7%–75.4%) compared to the overall population of respondents (22.9%). A previous study showed that patients with chronic conditions had distorted perceptions of their risk of severe COVID-19: about 20.0% did not feel at risk and could therefore adopt incautious attitudes.^40^ However, this study was published in 2020 and it is possible that risk perception in these patients evolved in view of the information collected along the pandemic.

In the present study, only 10 patients were hospitalized for COVID-19 which makes any analysis of risk factors impossible. It is well-established that AR (at all ages) and asthma (in patients aged <65) act as protective factors against COVID-19 infection,^41^ and that asthma is not over-represented in hospitalized patients with severe pneumonia due to COVID-19 infection.^42^ Furthermore, the use of inhaled corticosteroids is safe in asthma patients with COVID-19 infection, and it has been proposed that inhaled corticosteroids may confer some degree of protection against COVID-19 infection and the development of severe disease. In contrast, chronic or recurrent use of systemic corticosteroids before COVID-19 infection is a major risk factor of poor outcomes and worst survival in asthma patients.^42^ However, in our population of allergic patients treated with SLIT, it is very unlikely that there will be patients with chronic or recurrent use of systemic corticosteroids.

At the end of November 2021, 77.1% of the total French population had received at least 1 dose of COVID-19 vaccine and 75.5% were fully vaccinated (people that received 2 injections).^43^ In our survey, 90.0% of the patients declared being vaccinated for COVID-19. Since vaccination, 81.1% of the patients felt no longer or just a little at risk from COVID-19. Suffering from an allergic disease did not make patients feeling more vulnerable to side effects of COVID-19 vaccine for 79.6% of them. Current reports and recommendations do not suggest any relevant interference compromising neither the safety nor the efficacy of AIT, biologicals or COVID-19 vaccines.^16^^,^^44^ From a mechanistic point of view, AIT and COVID-19 immune responses do not seem to interfere negatively. Patients treated with AIT might even benefit by rebalancing the innate immune system and favoring protective responses. No data on the effects of AIT on the COVID-19 vaccine-induced antibody production are available, but due to different antigen specificity, it can be speculated that there is no interference. Current recommendations indicate that COVID-19 vaccines should be administered after an interval of 7 days from the subcutaneous allergy vaccines to unequivocally assign potential side effect of each one.^44^ Likewise, sublingual daily doses should be stopped 3 days before vaccine administration and restarted 7 days after.^45^ Further evidence from disease registries and other real-world data bases must be accumulated in order to refine the recommendations.

This study has some limitations that must be considered when interpreting the data. First, the study design had inherent limitations in terms of susceptibility to bias and confounding. As it was cross-sectional, it might not represent the accurate evaluation of the impact of COVID-19 on patients with allergy treated with SLIT along all the pandemic course, and also further restricting any causality assessment. However, since the analyses were restricted to descriptive statistics, such risks had been limited and no over-interpretation in terms of causality had been made. Second, the survey was only based on patient feedback not validated by a physician and could therefore create some biases related to patients’ responses and interpretation. Finally, the population of respondents was not representative of the Stallergenes-NPP database regarding age, gender and regions of residence. Nevertheless, the responses given by the participants reflected what is observed in the global population by French physicians and allergists. Despite the limitations, this patient survey met the targeted sample size and reach enough precision to be confident in the conclusions. The results of the survey were generally aligned with those of other observational studies regarding allergic disease management and perception of COVID-19 infection during the pandemic.^3^^,^^28^^,^^30^

## Conclusions

This French survey showed that, during the period of COVID-19 pandemic (started in 2020 and ongoing in 2021 with a lowest incidence rate of COVID-19 at the time of the survey), patients under SLIT reflected quite well the global population in terms of allergy characteristics, allergic disease management and perception of COVID-19 infection. Most respondents were poly-allergic and experienced moderate to severe AR and had a persistent AR profile, with a duration of disease of >15 years. Importantly, most of the patients could differentiate between allergy and COVID-19 symptoms. In terms of management of their allergic disease, more than half of the patients reported being able to consult (mainly through physical consultation) a physician as much as desired during lockdown. Overall, very few patients reported having been infected with COVID-19 since the beginning of the pandemic. The results also showed that the SLIT did not have any negative impact on the COVID-19 symptoms. The present cross-sectional survey provides an accurate picture of what was experienced and perceived by patients with allergy under SLIT during the COVID-19 pandemic in 2020/2021 in France.

## Abbreviations

AIT, allergen immunotherapy; AR, allergic rhinitis; ARIA, Allergic Rhinitis and its Impact on Asthma; COVID-19, coronavirus disease 2019; HCPs, healthcare professionals; HDM, house dust mites; NPP, Named-Patient Products; SLIT, sublingual immunotherapy.

## Funding

This work was funded by Stallergenes Greer.

## Availability of data and materials

Data can be available on reasonable justified request to the corresponding author.

## Author's contributions

Study design and data interpretation: PD, SS, CR, GWC, PD; All authors revised and approved the final manuscript.

## Ethics approval

As data reported in this study derives from a survey, and following the French Data Protection law, no submission of the study protocol to the opinion of an Ethics Committee or authorization from the French Health Authority (ANSM) was required.

## Author's consent for publication

All authors approved the publication of this manuscript.

## Declaration of competing interest

PD reports personal support by Stallergenes Greer for the present manuscript; receiving consulting fees from ALK-Abello, Astra Zeneca, Boehringer Ingelheim, Chiesi, GlaxoSmithKline, Viatris, Menarini, and Stallergenes Greer; payments or honoraria for lectures, presentations, speakers bureaus, manuscript writing or educational events from ALK-Abello, Astra Zeneca, Boehringer Ingelheim, Chiesi, GlaxoSmithKline, Viatris, Menarini, Novartis, Procter & Gamble, and Stallergenes Greer; and support for attending meetings and/or travel from ALK-Abello, Astra Zeneca, Boehringer Ingelheim, Chiesi, GlaxoSmithKline, Viatris, Menarini, Novartis, Procter & Gamble, and Stallergenes Greer. SS reports payment or honoraria for lectures, presentations, speakers bureaus, manuscript writing or educational events from DBV Technologies, Thermo Fisher Scientific, and ALK-Greer; and support for attending meetings and/or travel from Sanofi, ALK-Greer, and Stallergenes Greer. CR reports support by Stallergenes Greer (to Asthme & Allergies) for the present manuscript; grants and contracts by ALK, AstraZeneca, GSK, and Chiesi; payment or honoraria for lectures, presentations, speakers bureaus, manuscript writing or educational events from Stallergenes Greer, ALK, AstraZeneca, GSK, and Novartis; and support for attending meetings and/or travel from Stallergenes Greer, ALK, and GSK. GWC reports receiving consulting fees from AstraZeneca, Glaxo Smith Kline, Hal Allergy, Menarini, Sanofi, and Stallergenes; payment or honoraria for lectures, presentations, speakers bureaus, manuscript writing or educational events from AstraZeneca, Glaxo Smith Kline, Hal Allergy, Menarini, Sanofi, and Stallergenes; and support for attending meetings and/or travel by AstraZeneca, Glaxo Smith Kline, Hal Allergy, Menarini, Sanofi, and Stallergenes. PD reports receiving medical writing support from Stallergenes Greer for the present manuscript; institutional grants or contracts from ALK-Abelló, AstraZeneca, GlaxoSmithKline, Menarini, Puressentiel, Stallergenes Greer, ThermoFisher Scientific, Viatris; and support for attending meetings and/or travel from ALK and Stallergenes Greer.
