# Supplementary figures and images for: Respiratory allergic diseases and allergen immunotherapy: A French patient survey before and during the COVID-19 pandemic
Source: World Allergy Organ J. 2024 Apr 10;17(4):100902. doi: 10.1016/j.waojou.2024.100902 (PMC11017353; doi:10.1016/j.waojou.2024.100902)

**APPENDIX A. e-Questionnaire**


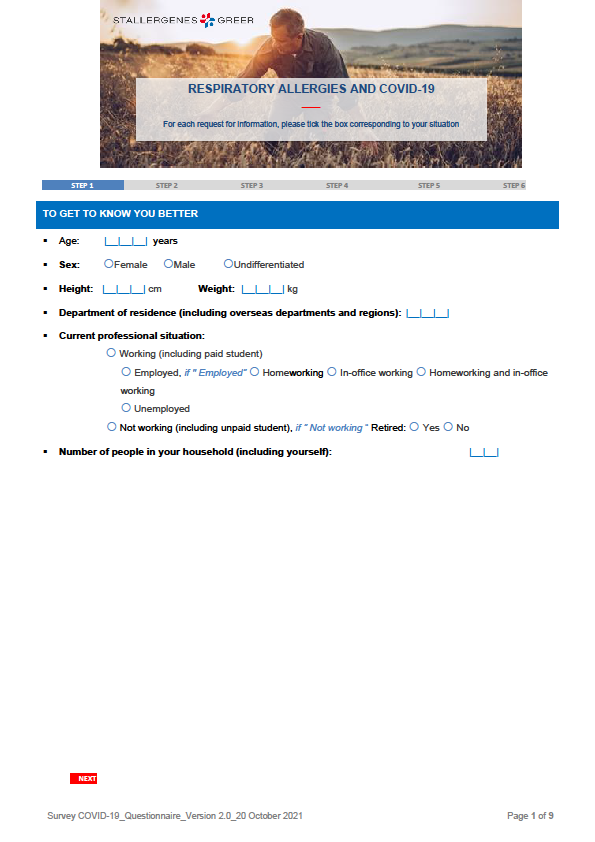


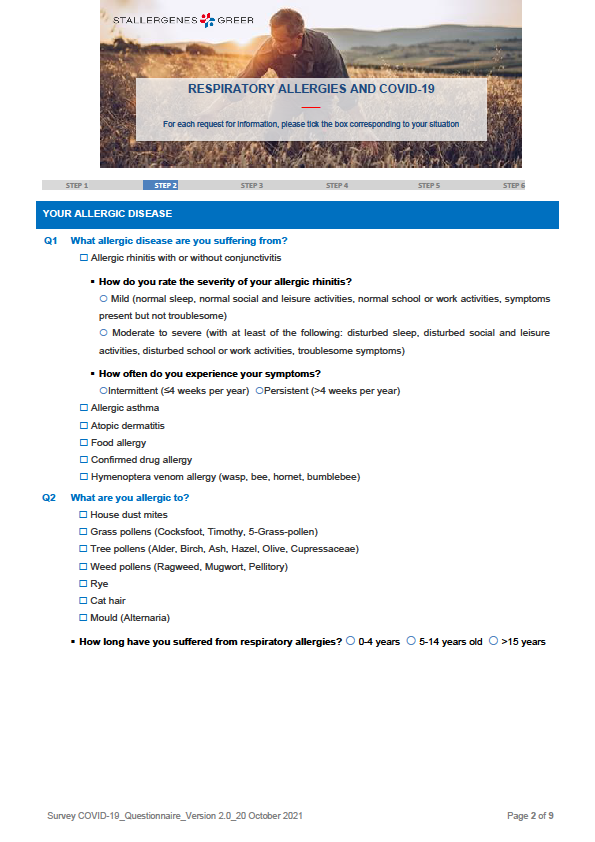


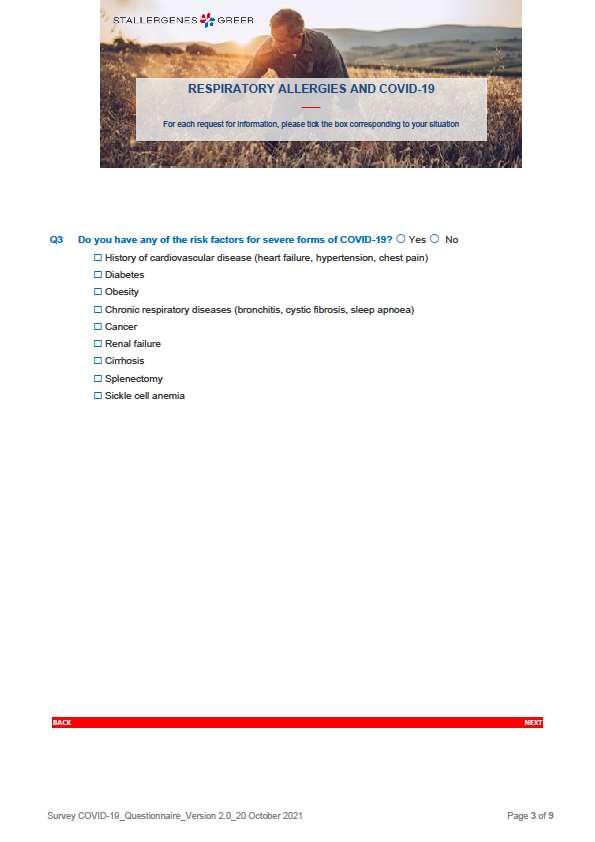


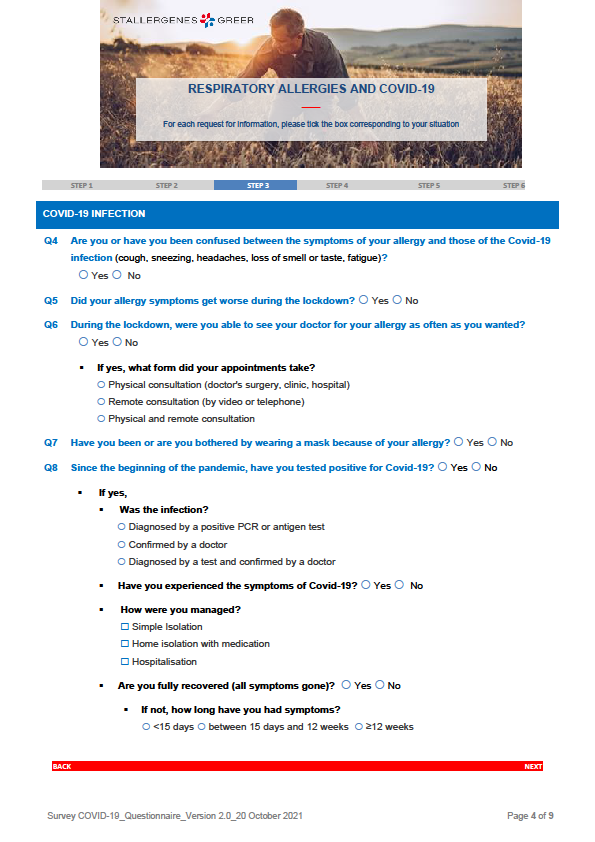


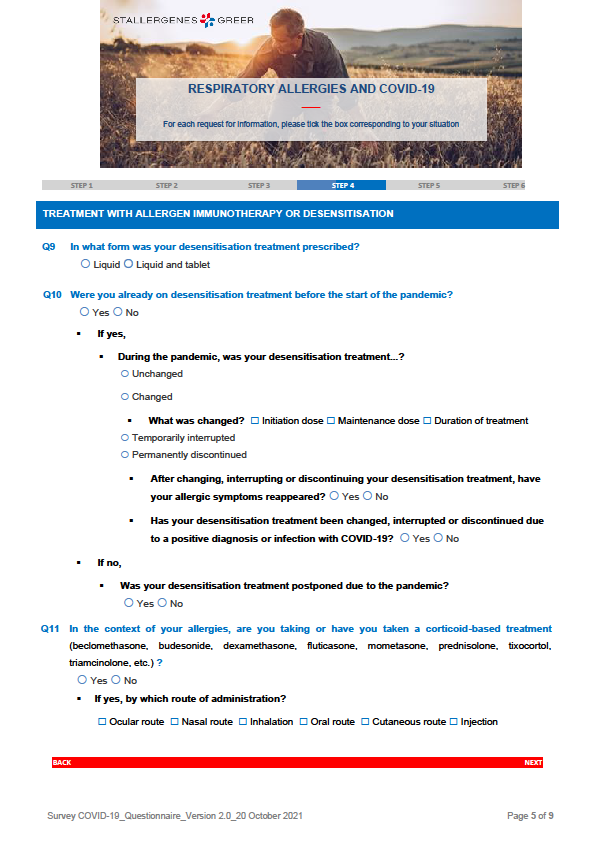


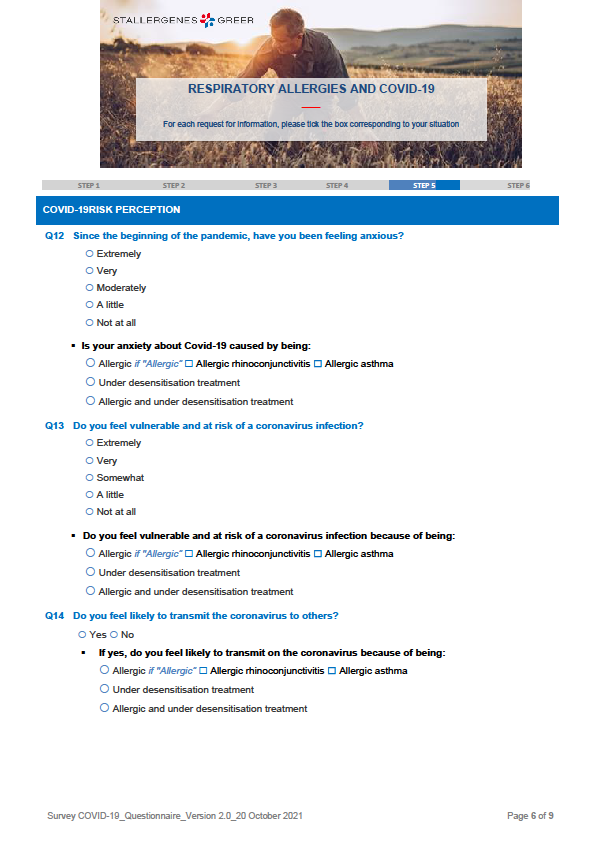


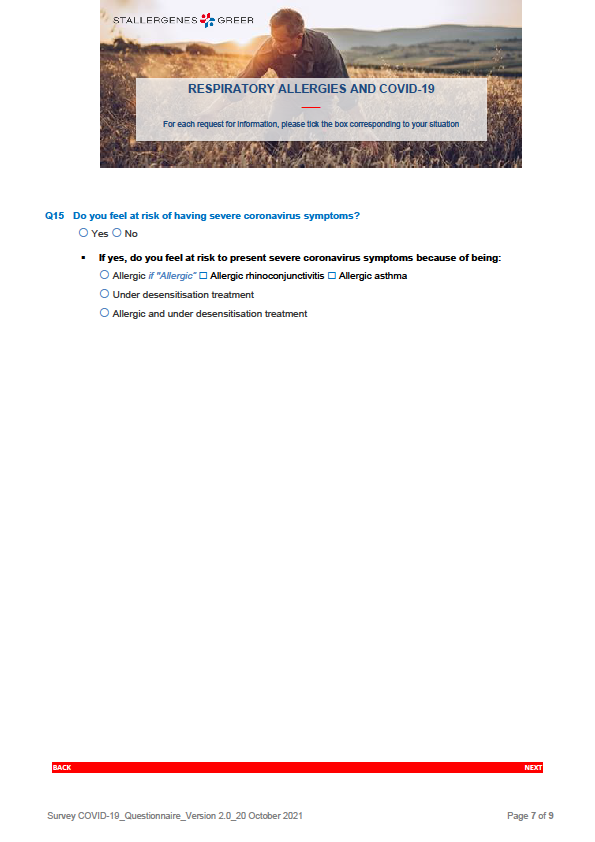


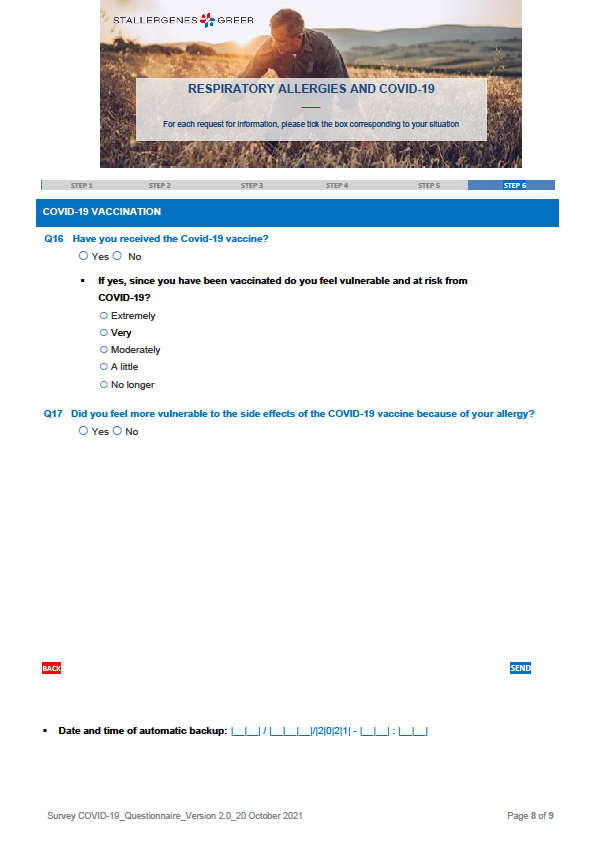

Supplement: Multimedia component 1 [file mmc1.docx]
