# Supplementary material for: Respiratory allergic diseases and allergen immunotherapy: A French patient survey before and during the COVID-19 pandemic
Source: World Allergy Organ J. 2024 Apr 10;17(4):100902. doi: 10.1016/j.waojou.2024.100902 (PMC11017353; doi:10.1016/j.waojou.2024.100902)
Supplement: Multimedia component 2 [file mmc2.docx]

**APPENDIX B. Supplementary data**

**Table S1.** Region of residence of the participants in the survey.

| **Region** | **N=3,662** |
| --- | --- |
| Auvergne-Rhône-Alpes | 464 (12.7) |
| Bourgogne-Franche-Comté | 145 (4.0) |
| Bretagne | 122 (3.3) |
| Centre-Val-de-Loire | 123 (3.4) |
| Corse | 11 (0.3) |
| Grand Est | 318 (8.7) |
| Hauts-de-France | 246 (6.7) |
| Ile-de-France | 796 (21.7) |
| Normandie | 133 (3.6) |
| Nouvelle-Aquitaine | 280 (7.6) |
| Occitanie | 379 (10.3) |
| Pays de la Loire | 235 (6.4) |
| Provence-Alpes-Côte d’Azur^1^ | 359 (9.8) |
| DOM-TOM^2^ | 51 (1.4) |

All data are shown as n (%)

^1^ Includes Monaco.

^2^ DOM-TOM includes Guadeloupe, Martinique, Guyane, La Réunion, Mayotte and unspecified overseas.

**Table S2.** Patient profile according to hospitalization status.

|  |  | **Infected hospitalized N = 10 (0.5%)** | **Infected non-hospitalized N = 537 (14.5%)** | **Non-infected with COVID-19 N = 3,159 (85.0%)** |
| --- | --- | --- | --- | --- |
| Age (years) | N (mv) | 10 | 518 (19) | 3,088 (71) |
|  | Mean ± SD | 42.10 ± 12.94 | 37.34 ± 12.38 | 39.61 ± 13.11 |
|  | Median | 44.00 | 35.00 | 39.00 |
|  | Q1 ; Q3 | 29.00 ; 53.00 | 28.00 ; 44.00 | 30.00 ; 48.00 |
|  | Min ; Max | 20.0 ; 57.0 | 18.0 ; 77.0 | 18.0 ; 88.0 |
| Allergic diseases | N | 10 | 537 | 3,159 |
|  | Allergic rhinitis with or without conjunctivitis | 8 (80.0%) | 437 (81.4%) | 2,524 (79.9%) |
|  | Allergic asthma | 4 (40.0%) | 224 (41.7%) | 1,217 (38.5%) |
|  | Atopic dermatitis | 3 (30.0%) | 52 (9.7%) | 295 (9.3%) |
|  | Food allergy | 2 (20.0%) | 101 (18.8%) | 439 (13.9%) |
|  | Confirmed drug allergy | 0 | 26 (4.8%) | 142 (4.5%) |
|  | Hymenoptera venom allergy (wasp, bee, hornet, bumblebee) | 2 (20.0%) | 20 (3.7%) | 101 (3.2%) |
| Severity of allergic rhinitis | N (mv) | 8 | 434 (3) | 2512 (12) |
|  | Mild | 1 (12.5%) | 168 (38.7%) | 1,071 (42.6%) |
|  | Moderate to severe | 7 (87.5%) | 266 (61.3%) | 1,441 (57.4%) |
| ARIA classification (revised 2010) of allergic rhinitis | N (mv) | 8 | 427 (10) | 2497 (27) |
|  | Intermittent (≤4 weeks per year) | 3 (37.5%) | 118 (27.6%) | 746 (29.9%) |
|  | Persistent (>4 weeks per year) | 5 (62.5%) | 309 (72.4%) | 1,751 (70.1%) |
| ARIA classification of allergic rhinitis:  frequency*severity [a] | N (mv) | 8 | 425 (12) | 2488 (36) |
|  | Intermittent mild | 1 (12.5%) | 67 (15.8%) | 471 (18.9%) |
|  | Persistent mild | 0 | 94 (22.1%) | 588 (23.6%) |
|  | Intermittent moderate to severe | 2 (25.0%) | 50 (11.8%) | 273 (11.0%) |
|  | Persistent moderate to severe | 5 (62.5%) | 214 (50.4%) | 1,156 (46.5%) |
| Allergen families | N | 10 | 537 | 3159 |
|  | House dust mites | 6 (60.0%) | 327 (60.9%) | 1,963 (62.1%) |
|  | Grass pollens (cocksfoot, timothy, 5-Grass pollen and rye) | 5 (50.0%) | 330 (61.5%) | 1,942 (61.5%) |
|  | Tree pollens (alder, birch, ash, hazel, olive, cupressaceae) | 7 (70.0%) | 322 (60.0%) | 1,813 (57.4%) |
|  | Weed pollens (ragweed, mugwort, pellitory) | 3 (30.0%) | 114 (21.2%) | 719 (22.8%) |
|  | Cat dander | 2 (20.0%) | 212 (39.5%) | 1,167 (36.9%) |
|  | Mould (alternaria) | 4 (40.0%) | 98 (18.2%) | 528 (16.7%) |
| Allergic profile | N (mv) | 10 | 535 (2) | 3,156 (3) |
|  | Mono-allergic | 5 (50.0%) | 139 (26.0%) | 867 (27.5%) |
|  | Poly-allergic | 5 (50.0%) | 396 (74.0%) | 2,289 (72.5%) |
| Seniority of allergic respiratory disease | N (mv) | 10 | 532 (5) | 3,120 (39) |
|  | [0-4] years | 0 | 62 (11.7%) | 338 (10.8%) |
|  | [5-14] years | 3 (30.0%) | 204 (38.3%) | 1,119 (35.9%) |
|  | ≥15 years | 7 (70.0%) | 266 (50.0%) | 1,663 (53.3%) |
| Criteria of severe COVID-19 infection risk | N (mv) | 10 | 537 | 3,154 (5) |
|  | No | 8 (80.0%) | 492 (91.6%) | 2,884 (91.4%) |
|  | Yes | 2 (20.0%) | 45 (8.4%) | 270 (8.6%) |
| If Yes, SOC classification for comorbidities | N (mv) | 2 | 39 (6) | 261 (9) |
|  | Cardiac disorders | 1 (50.0%) | 11 (24.4%) | 64 (23.7%) |
|  | Metabolism and nutrition disorders | 1 (50.0%) | 23 (51.1%) | 166 (61.5%) |
|  | Respiratory, thoracic and mediastinal disorders | 0 | 19 (42.2%) | 96 (35.6%) |
|  | Neoplasms benign, malignant and unspecified | 0 | 2 (4.4%) | 13 (4.8%) |
|  | Renal and urinary disorders | 0 | 0 | 1 (0.4%) |
|  | Hepatobiliary disorders | 0 | 0 | 1 (0.4%) |
|  | Surgical and medical procedures | 0 | 0 | 2 (0.7%) |
|  | Congenital, familial and genetic disorders | 0 | 0 | 0 |
| Data are n (%); mv = missing values  SOC: System Organ Class  [a] Mild: normal sleep, normal social and leisure activities, normal school or work activities and symptoms present but not troublesome; Moderate to severe (with at least one sign): disturbed sleep, disturbed social and leisure activities, disturbed school or work activities, troublesome symptoms | | | | |

**Table S3.** Patient profile according to COVID-19 status and duration of symptoms*

|  | | **COVID-19-infected patients with long duration of symptoms (Long COVID) N = 85 (2.3%)** | **COVID-19-infected patients with short duration of symptoms N = 462 (12.4%)** | **Non-infected patients with COVID-19 N = 3,159 (84.9%)** |
| --- | --- | --- | --- | --- |
| Age (years) | N (mv) | 81 (4) | 447 (15) | 3,088 (71) |
|  | Mean ± SD | 38.16 ± 11.72 | 37.30 ± 12.52 | 39.61 ± 13.11 |
|  | Median | 37.00 | 35.00 | 39.00 |
|  | Q1 ; Q3 | 29.00 ; 45.00 | 28.00 ; 44.00 | 30.00 ; 48.00 |
|  | Min ; Max | 18.0 ; 66.0 | 18.0 ; 77.0 | 18.0 ; 88.0 |
| Allergic diseases | N | 85 | 462 | 3159 |
|  | Allergic rhinitis with or without conjunctivitis | 69 (81.2%) | 376 (81.4%) | 2,524 (79.9%) |
|  | Allergic asthma | 41 (48.2%) | 187 (40.5%) | 1,217 (38.5%) |
|  | Atopic dermatitis | 17 (20.0%) | 38 (8.2%) | 295 (9.3%) |
|  | Food allergy | 32 (37.6%) | 71 (15.4%) | 439 (13.9%) |
|  | Confirmed drug allergy | 6 (7.1%) | 20 (4.3%) | 142 (4.5%) |
|  | Hymenoptera venom allergy (wasp, bee, hornet, bumblebee) | 7 (8.2%) | 15 (3.2%) | 101 (3.2%) |
| Severity of allergic rhinitis | N (mv) | 68 (1) | 374 (2) | 2,512 (12) |
|  | Mild | 13 (19.1%) | 156 (41.7%) | 1,071 (42.6%) |
|  | Moderate to severe | 55 (80.9%) | 218 (58.3%) | 1,441 (57.4%) |
| ARIA classification (revised 2010) of allergic rhinitis | N (mv) | 68 (1) | 367 (9) | 2,497 (27) |
|  | Intermittent (≤ 4 weeks per year) | 11 (16.2%) | 110 (30.0%) | 746 (29.9%) |
|  | Persistent (> 4 weeks per year) | 57 (83.8%) | 257 (70.0%) | 1,751 (70.1%) |
| ARIA classification of allergic rhinitis: frequency*severity [a] | N (mv) | 67 (2) | 366 (10) | 2,488 (36) |
|  | Intermittent mild | 1 (1.5%) | 67 (18.3%) | 471 (18.9%) |
|  | Persistent mild | 11 (16.4%) | 83 (22.7%) | 588 (23.6%) |
|  | Intermittent moderate to severe | 9 (13.4%) | 43 (11.7%) | 273 (11.0%) |
|  | Persistent moderate to severe | 46 (68.7%) | 173 (47.3%) | 1,156 (46.5%) |
| Allergen families | N | 85 | 462 | 3,159 |
|  | House dust mites | 56 (65.9%) | 277 (60.0%) | 1,963 (62.1%) |
|  | Grass pollens (cocksfoot, timothy, 5-Grass pollen and rye) | 59 (69.4%) | 276 (59.7%) | 1,942 (61.5%) |
|  | Tree pollens (alder, birch, ash, hazel, olive, cupressaceae) | 57 (67.1%) | 272 (58.9%) | 1,813 (57.4%) |
|  | Weed pollens (ragweed, mugwort, pellitory) | 27 (31.8%) | 90 (19.5%) | 719 (22.8%) |
|  | Cat dander | 44 (51.8%) | 170 (36.8%) | 1,167 (36.9%) |
|  | Mould (alternaria) | 19 (22.4%) | 83 (18.0%) | 528 (16.7%) |
| Allergic profile | N (mv) | 85 | 460 (2) | 3,156 (3) |
|  | Mono-allergic | 13 (15.3%) | 131 (28.5%) | 867 (27.5%) |
|  | Poly-allergic | 72 (84.7%) | 329 (71.5%) | 2,289 (72.5%) |
| Seniority of allergic respiratory disease | N (mv) | 85 | 457 (5) | 3,120 (39) |
|  | [0-4] years | 8 (9.4%) | 54 (11.8%) | 338 (10.8%) |
|  | [5-14] years | 28 (32.9%) | 179 (39.2%) | 1,119 (35.9%) |
|  | ≥ 15 years | 49 (57.6%) | 224 (49.0%) | 1,663 (53.3%) |
| Criteria of severe COVID-19 infection risk | N (mv) | 85 | 462 | 3,154 (5) |
|  | No | 70 (82.4%) | 430 (93.1%) | 2,884 (91.4%) |
|  | Yes | 15 (17.6%) | 32 (6.9%) | 270 (8.6%) |
| If Yes, SOC classification for comorbidities | N (mv) | 12 (3) | 29 (3) | 261 (9) |
|  | Cardiac disorders | 2 (13.3%) | 10 (31.3%) | 64 (23.7%) |
|  | Metabolism and nutrition disorders | 8 (53.3%) | 16 (50.0%) | 166 (61.5%) |
|  | Respiratory, thoracic and mediastinal disorders | 8 (53.3%) | 11 (34.4%) | 96 (35.6%) |
|  | Neoplasms benign, malignant and unspecified | 0 | 2 (6.3%) | 13 (4.8%) |
|  | Renal and urinary disorders | 0 | 0 | 1 (0.4%) |
|  | Hepatobiliary disorders | 0 | 0 | 1 (0.4%) |
|  | Surgical and medical procedures | 0 | 0 | 2 (0.7%) |
|  | Congenital, familial and genetic disorders | 0 | 0 | 0 |

Data are n (%); mv = missing values

Due to rounding, total percentages may differ from 100.0%

SOC: System Organ Class

[a] Mild: normal sleep, normal social and leisure activities, normal school or work activities and symptoms present but not troublesome; Moderate to severe (with at least one sign): disturbed sleep, disturbed social and leisure activities, disturbed school or work activities, troublesome symptoms

*Long duration of symptoms: >12 weeks; Short duration of symptoms: <12 weeks

**Table S4.** Patient profile according to corticosteroid-based treatment uptake

|  | | **With  corticosteroid-based**  **treatment uptake N = 1,325 (35.6%)** | **With no  corticosteroid-based**  **treatment uptake N = 2,362 (63.4%)** |
| --- | --- | --- | --- |
| Age (years) | N (mv) | 1,291 (34) | 2,306 (56) |
|  | Mean ± SD | 40.16 ± 12.47 | 38.70 ± 13.24 |
|  | Median | 39.00 | 37.00 |
|  | Q1 ; Q3 | 31.00 ; 48.00 | 29.00 ; 47.00 |
|  | Min ; Max | 18.0 ; 80.0 | 18.0 ; 88.0 |
| Allergic diseases | N | 1,325 | 2,362 |
|  | Allergic rhinitis with or without conjunctivitis | 1,074 (81.1%) | 1,882 (79.7%) |
|  | Allergic asthma | 721 (54.4%) | 719 (30.4%) |
|  | Atopic dermatitis | 194 (14.6%) | 153 (6.5%) |
|  | Food allergy | 250 (18.9%) | 290 (12.3%) |
|  | Confirmed drug allergy | 83 (6.3%) | 84 (3.6%) |
|  | Hymenoptera venom allergy (wasp, bee, hornet, bumblebee) | 60 (4.5%) | 60 (2.5%) |
| Severity of allergic rhinitis | N (mv) | 1,070 (4) | 1,871 (11) |
|  | Mild | 359 (33.6%) | 878 (46.9%) |
|  | Moderate to severe | 711 (66.4%) | 993 (53.1%) |
| ARIA classification (revised 2010) of allergic rhinitis | N (mv) | 1,059 (15) | 1,860 (22) |
|  | Intermittent (≤4 weeks per year) | 263 (24.8%) | 600 (32.3%) |
|  | Persistent (>4 weeks per year) | 796 (75.2%) | 1,260 (67.7%) |
| ARIA classification of allergic rhinitis:  frequency*severity [a] | N (mv) | 1,056 (18) | 1,852 (30) |
|  | Intermittent mild | 150 (14.2%) | 388 (21.0%) |
|  | Persistent mild | 202 (19.1%) | 478 (25.8%) |
|  | Intermittent moderate to severe | 112 (10.6%) | 210 (11.3%) |
|  | Persistent moderate to severe | 592 (56.1%) | 776 (41.9%) |
| Allergen families | N | 1,325 | 2,362 |
|  | House dust mites | 880 (66.4%) | 1,409 (59.7%) |
|  | Grass pollens (cocksfoot, timothy, 5-Grass pollen and rye) | 849 (64.1%) | 1,418 (60.0%) |
|  | Tree pollens (alder, birch, ash, hazel, olive, cupressaceae) | 811 (61.2%) | 1,316 (55.7%) |
|  | Weed pollens (ragweed, mugwort, pellitory) | 338 (25.5%) | 495 (21.0%) |
|  | Cat dander | 565 (42.6%) | 813 (34.4%) |
|  | Mould (alternaria) | 286 (21.6%) | 339 (14.4%) |
| Allergic profile | N (mv) | 1,325 | 2,357 (5) |
|  | Mono-allergic | 280 (21.1%) | 725 (30.8%) |
|  | Poly-allergic | 1,045 (78.9%) | 1,632 (69.2%) |
| Seniority of allergic respiratory disease | N (mv) | 1,314 (11) | 2,329 (33) |
|  | [0-4] years | 111 (8.4%) | 285 (12.2%) |
|  | [5-14] years | 417 (31.7%) | 906 (38.9%) |
|  | ≥15 years | 786 (59.8%) | 1,138 (48.9%) |
| Criteria of severe COVID-19 infection risk | N (mv) | 1,322 (3) | 2,360 (2) |
|  | No | 1,164 (88.0%) | 2,204 (93.4%) |
|  | Yes | 158 (12.0%) | 156 (6.6%) |
| If Yes, SOC classification for comorbidities | N (mv) | 149 (9) | 150 (6) |
|  | Cardiac disorders | 28 (17.7%) | 47 (30.1%) |
|  | Metabolism and nutrition disorders | 92 (58.2%) | 97 (62.2%) |
|  | Respiratory, thoracic and mediastinal disorders | 72 (45.6%) | 42 (26.9%) |
|  | Neoplasms benign, malignant and unspecified | 6 (3.8%) | 9 (5.8%) |
|  | Renal and urinary disorders | 1 (0.6%) | 0 |
|  | Hepatobiliary disorders | 1 (0.6%) | 0 |
|  | Surgical and medical procedures | 0 | 2 (1.3%) |
|  | Congenital, familial and genetic disorders | 0 | 0 |

Data are n (%); mv = missing values

SOC: System Organ Class

[a] Mild: normal sleep, normal social and leisure activities, normal school or work activities and symptoms present but not troublesome; Moderate to severe (with at least one sign): disturbed sleep, disturbed social and leisure activities, disturbed school or work activities, troublesome symptoms

**Table S5.** COVID-19 risk perception.

|  | **N=3,723** |
| --- | --- |
| Feeling anxiety *(n=3,716)* |  |
| Extremely | 108 (2.9) |
| Very | 249 (6.7) |
| Moderately | 643 (17.3) |
| A little | 664 (17.9) |
| Not at all | 2,052 (55.2) |
| Feeling anxiety because of being *(n=1,664)* |  |
| Allergic | 818 (49.2) |
| Under desensitization treatment | 66 (4.0) |
| Allergic and under desensitization treatment | 214 (12.9) |
| No reason specified | 566 (34.0) |
| If allergic, type of allergy *(n=802)* |  |
| Allergic asthma | 462 (57.6) |
| Allergic rhinoconjunctivitis | 340 (42.4) |
| Feeling vulnerability *(n=3,709)* |  |
| Extremely | 69 (1.9) |
| Very | 245 (6.6) |
| Moderately | 656 (17.7) |
| A little | 770 (20.8) |
| Not at all | 1,969 (53.1) |
| Feeling vulnerability because of being *(n=1,740)* |  |
| Allergic | 1,031 (59.3) |
| Allergic and under desensitization treatment | 202 (11.6) |
| Under desensitization treatment | 55 (3.2) |
| No reason specified | 452 (26.0) |
| If allergic, type of allergy *(n=1,021)* |  |
| Allergic asthma | 645 (63.2) |
| Allergic rhinoconjunctivitis | 376 (36.8) |
| Feeling at risk to transmit the coronavirus *(n=3,709)* | 728 (19.6) |
| Feeling at risk to transmit the coronavirus because of being *(n=728)* |  |
| Allergic | 293 (40.2) |
| Allergic and under desensitization treatment | 56 (7.7) |
| Under desensitization treatment | 12 (1.6) |
| No reason specified | 367 (50.4) |
| If allergic, type of allergy *(n=289)* |  |
| Allergic asthma | 127 (43.9) |
| Allergic rhinoconjunctivitis | 162 (56.1) |
| Feeling at risk to present severe symptoms of coronavirus *(n=3,701)* | 846 (22.9) |
| Feeling at risk to present severe symptoms of coronavirus because of being *(n=846)* |  |
| Allergic | 637 (75.3) |
| Allergic and under desensitization treatment | 96 (11.3) |
| Under desensitization treatment | 2 (0.2) |
| No reason specified | 111 (13.1) |
| If allergic, type of allergy *(n=634)* |  |
| Allergic asthma | 498 (78.5) |
| Allergic rhinoconjunctivitis | 136 (21.5) |

All values are n (%). N, number of patients in the main analysis; n, number of patients with data.

**Table S6.** COVID-19 risk perception according to obesity.

|  | | **Not obese patients N = 3,570 (95.9%)** | **Obese patients N = 153 (4.1%)** |
| --- | --- | --- | --- |
| Feeling anxiety | N (mv) | 3,563 (7) | 153 |
|  | Extremely | 101 (2.8%) | 7 (4.6%) |
|  | Very | 229 (6.4%) | 20 (13.1%) |
|  | Moderately | 601 (16.9%) | 42 (27.5%) |
|  | A little | 632 (17.7%) | 32 (20.9%) |
|  | Not at all | 2,000 (56.1%) | 52 (34.0%) |
| Feeling anxiety because of being | N | 1,563 | 101 |
|  | Allergic | 759 (48.6%) | 59 (58.4%) |
|  | Under desensitization treatment | 64 (4.1%) | 2 (2.0%) |
|  | Allergic and under desensitization treatment | 204 (13.1%) | 10 (9.9%) |
|  | No reasons specified | 536 (34.3%) | 30 (29.7%) |
| If allergic, type of allergy | N (mv) | 743 (16) | 59 |
|  | Allergic asthma | 417 (56.1%) | 45 (76.3%) |
|  | Allergic rhinoconjunctivitis | 326 (43.9%) | 14 (23.7%) |
| Feeling vulnerability | N (mv) | 3,556 (14) | 153 |
|  | Extremely | 58 (1.6%) | 11 (7.2%) |
|  | Very | 221 (6.2%) | 24 (15.7%) |
|  | Moderately | 611 (17.2%) | 45 (29.4%) |
|  | A little | 742 (20.9%) | 28 (18.3%) |
|  | Not at all | 1,924 (54.1%) | 45 (29.4%) |
| Feeling vulnerability because of being | N | 1,632 | 108 |
|  | Allergic | 964 (59.1%) | 67 (62.0%) |
|  | Under desensitization treatment | 54 (3.3%) | 1 (0.9%) |
|  | Allergic and under desensitization treatment | 187 (11.5%) | 15 (13.9%) |
|  | No reasons specified | 427 (26.2%) | 25 (23.1%) |
| If allergic, type of allergy | N (mv) | 954 (10) | 67 |
|  | Allergic asthma | 594 (62.3%) | 51 (76.1%) |
|  | Allergic rhinoconjunctivitis | 360 (37.7%) | 16 (23.9%) |
| Feeling at risk to transmit the coronavirus | N (mv) | 3,556 (14) | 153 |
|  | No | 2,877 (80.9%) | 104 (68.0%) |
|  | Yes | 679 (19.1%) | 49 (32.0%) |
| Feeling at risk to transmit the coronavirus because of being | N | 679 | 49 |
|  | Allergic | 272 (40.1%) | 21 (42.9%) |
|  | Under desensitization treatment | 11 (1.6%) | 1 (2.0%) |
|  | Allergic and under desensitization treatment | 52 (7.7%) | 4 (8.2%) |
|  | No reasons specified | 344 (50.7%) | 23 (46.9%) |
| If allergic, type of allergy | N (mv) | 268 (4) | 21 |
|  | Allergic asthma | 117 (43.7%) | 10 (47.6%) |
|  | Allergic rhinoconjunctivitis | 151 (56.3%) | 11 (52.4%) |
| Feeling at risk to present severe symptoms of coronavirus | N | 3,548 (22) | 153 |
|  | No | 2,794 (78.7%) | 61 (39.9%) |
|  | Yes | 754 (21.3%) | 92 (60.1%) |
| Feeling at risk to present severe symptoms of coronavirus because of being | N | 754 | 92 |
|  | Allergic | 575 (76.3%) | 62 (67.4%) |
|  | Under desensitization treatment | 2 (0.3%) | 0 (0.0%) |
|  | Allergic and under desensitization treatment | 87 (11.5%) | 9 (9.8%) |
|  | No reasons specified | 90 (11.9%) | 21 (22.8%) |
| If allergic, type of allergy | N (mv) | 572 (3) | 62 |
|  | Allergic asthma | 449 (78.5%) | 49 (79.0%) |
|  | Allergic rhinoconjunctivitis | 123 (21.5%) | 13 (21.0%) |

mv = missing values; Obese patients: patients with a BMI > 30 kg/m² and who ticked 'Obesity' as comorbidity.

**Table S7.** AIT treatment according to age classes.

|  | | **[18 - 29] years N = 903 (24.9%)** | **[30 - 49] years N = 1,964**  **(54.1%)** | **[50 - 64] years N = 598 (16.5%)** | **[65 - 74] years N = 145 (4.0%)** | **≥ 75 years N = 22 (0.6%)** |
| --- | --- | --- | --- | --- | --- | --- |
| Type of AIT treatment | N (mv) | 901 (2) | 1,958 (6) | 592 (6) | 142(3) | 21 (1) |
|  | Liquid | 698 (77.5%) | 1,540 (78.7%) | 479 (80.9%) | 118 (83.1%) | 13 (61.9%) |
|  | Liquid and tablet | 203 (22.5%) | 418 (21.3%) | 113 (19.1%) | 24 (16.9%) | 8 (38.1%) |
| Initiation of AIT before pandemic | N (mv) | 900 (3) | 1,960 (4) | 593 (5) | 143 (2) | 21 (1) |
|  | No | 35 (3.9%) | 61 (3.1%) | 13 (2.2%) | 2 (1.4%) | 0 |
|  | Yes | 865 (96.1%) | 1,899 (96.9%) | 580 (97.8%) | 141 (98.6%) | 21 (100.0%) |
| If initiation of AIT before pandemic, modification of treatment during pandemic | N (mv) | 864 (1) | 1,895 (4) | 579 (1) | 140 (1) | 21 (0) |
|  | Unchanged | 648 (75.0%) | 1,483 (78.3%) | 477 (82.4%) | 115 (82.1%) | 19 (90.5%) |
|  | Changed | 17 (2.0%) | 36 (1.9%) | 12 (2.1%) | 3 (2.1%) | 0 |
|  | Temporary interruption | 54 (6.3%) | 133 (7.0%) | 35 (6.0%) | 6 (4.3%) | 1 (4.8%) |
|  | Permanently discontinued | 145 (16.8%) | 243 (12.8%) | 55 (9.5%) | 16 (11.4%) | 1 (4.8%) |
| If treatment changed, type of modification | N | 17 | 36 | 12 | 3 | 0 |
|  | Initiation dose | 6 (35.3%) | 9 (25.0%) | 2 (16.7%) | 1 (33.3%) | 0 |
|  | Maintenance dose | 11 (64.7%) | 21 (58.3%) | 9 (75.0%) | 2 (66.7%) | 0 |
|  | Duration of treatment | 0 | 6 (16.7%) | 1 (8.3%) | 0 | 0 |
| If treatment changed, temporary interrupted or permanently discontinued, reappearance of allergic symptoms | N (mv) | 215 (1) | 411 (1) | 102 (0) | 25 (0) | 2 (0) |
|  | No | 67 (31.2%) | 153 (37.2%) | 44 (43.1%) | 13 (52.0%) | 1 (50.0%) |
|  | Yes | 148 (68.8%) | 258 (62.8%) | 58 (56.9%) | 12 (48.0%) | 1 (50.0%) |
| If treatment changed, temporary interrupted or permanently discontinued, caused by COVID-19 infection or positive diagnosis | N | 216 | 412 | 102 | 25 | 2 |
|  | No | 207 (95.8%) | 396 (96.1%) | 98 (96.1%) | 23 (92.0%) | 1 (50.0%) |
|  | Yes | 9 (4.2%) | 16 (3.9%) | 4 (3.9%) | 2 (8.0%) | 1 (50.0%) |
| If no initiation of AIT before pandemic, postponement of treatment because of pandemic | N (mv) | 29 (6) | 50 (11) | 10 (3) | 0 (2) | 0 (0) |
|  | No | 0 | 0 | 0 | 0 | 0 |
|  | Yes | 29 (100.0%) | 50 (100.0%) | 10 (100.0%) | 0 | 0 |
| Corticosteroid-based treatment uptake | N (mv) | 896 (7) | 1,952 (12) | 586 (12) | 142 (3) | 21 (1) |
|  | No | 630 (70.3%) | 1,202 (61.6%) | 371 (63.3%) | 88 (62.0%) | 15 (71.4%) |
|  | Yes | 266 (29.7%) | 750 (38.4%) | 215 (36.7%) | 54 (38.0%) | 6 (28.6%) |
| Data are n(%); mv = missing values  Due to rounding, total percentages may differ from 100.0% | | | | | | |

**Table S8.** COVID-19 risk perception according to age classes.

|  | | **[18 - 29] years N = 903 (24.9%)** | **[30 - 49] years N = 1,964**  **(54.1%)** | **[50 - 64] years N = 598 (16.5%)** | **[65 - 74] years N = 145 (4.0%)** | **≥ 75 years N = 22 (0.6%)** |
| --- | --- | --- | --- | --- | --- | --- |
| Feeling anxiety | N (mv) | 902 (1) | 1,962 (2) | 596 (2) | 143 (2) | 22 (0) |
|  | Extremely | 23 (2.5%) | 68 (3.5%) | 11 (1.8%) | 4 (2.8%) | 0 |
|  | Very | 66 (7.3%) | 147 (7.5%) | 24 (4.0%) | 7 (4.9%) | 1 (4.5%) |
|  | Moderately | 133 (14.7%) | 363 (18.5%) | 106 (17.8%) | 19 (13.3%) | 4 (18.2%) |
|  | A little | 128 (14.2%) | 334 (17.0%) | 142 (23.8%) | 38 (26.6%) | 4 (18.2%) |
|  | Not at all | 552 (61.2%) | 1050 (53.5%) | 313 (52.5%) | 75 (52.4%) | 13 (59.1%) |
| Feeling anxiety because of being | N | 350 | 912 | 283 | 68 | 9 |
|  | Allergic | 166 (47.4%) | 439 (48.1%) | 154 (54.4%) | 31 (45.6%) | 5 (55.6%) |
|  | Under desensitization treatment | 29 (8.3%) | 30 (3.3%) | 5 (1.8%) | 1 (1.5%) | 0 |
|  | Allergic and under desensitization treatment | 37 (10.6%) | 122 (13.4%) | 41 (14.5%) | 9 (13.2%) | 1 (11.1%) |
|  | No reason specified | 118 (33.7%) | 321 (35.2%) | 83 (29.3%) | 27 (39.7%) | 3 (33.3%) |
| If allergic, type of allergy | N (mv) | 164 (2) | 431 (8) | 149 (5) | 30 (1) | 5 (0) |
|  | Allergic asthma | 89 (54.3%) | 252 (58.5%) | 86 (57.7%) | 19 (63.3%) | 1 (20.0%) |
|  | Allergic rhinoconjunctivitis | 75 (45.7%) | 179 (41.5%) | 63 (42.3%) | 11 (36.7%) | 4 (80.0%) |
| Feeling vulnerability | N (mv) | 898 (5) | 1,961 (3) | 596 (2) | 141 (4) | 22 (0) |
|  | Extremely | 11 (1.2%) | 39 (2.0%) | 14 (2.3%) | 4 (2.8%) | 1 (4.5%) |
|  | Very | 44 (4.9%) | 143 (7.3%) | 46 (7.7%) | 8 (5.7%) | 1 (4.5%) |
|  | Moderately | 140 (15.6%) | 347 (17.7%) | 115 (19.3%) | 30 (21.3%) | 4 (18.2%) |
|  | A little | 152 (16.9%) | 403 (20.6%) | 144 (24.2%) | 41 (29.1%) | 7 (31.8%) |
|  | Not at all | 551 (61.4%) | 1029 (52.5%) | 277 (46.5%) | 58 (41.1%) | 9 (40.9%) |
| Feeling vulnerability because of being | N | 347 | 932 | 319 | 83 | 13 |
|  | Allergic | 226 (65.1%) | 546 (58.6%) | 185 (58.0%) | 40 (48.2%) | 8 (61.5%) |
|  | Under desensitization treatment | 13 (3.7%) | 25 (2.7%) | 9 (2.8%) | 5 (6.0%) | 1 (7.7%) |
|  | Allergic and under desensitization treatment | 34 (9.8%) | 115 (12.3%) | 37 (11.6%) | 11 (13.3%) | 1 (7.7%) |
|  | No reason specified | 74 (21.3%) | 246 (26.4%) | 88 (27.6%) | 27 (32.5%) | 3 (23.1%) |
| If allergic, type of allergy | N (mv) | 225 (1) | 540 (6) | 182 (3) | 40 (0) | 8 (0) |
|  | Allergic asthma | 149 (66.2%) | 338 (62.6%) | 112 (61.5%) | 26 (65.0%) | 2 (25.0%) |
|  | Allergic rhinoconjunctivitis | 76 (33.8%) | 202 (37.4%) | 70 (38.5%) | 14 (35.0%) | 6 (75.0%) |
| Feeling at risk to transmit the coronavirus | N (mv) | 901 (2) | 1958 (6) | 595 (3) | 144 (1) | 22 (0) |
|  | No | 731 (81.1%) | 1,532 (78.2%) | 495 (83.2%) | 126 (87.5%) | 21 (95.5%) |
|  | Yes | 170 (18.9%) | 426 (21.8%) | 100 (16.8%) | 18 (12.5%) | 1 (4.5%) |
| Feeling at risk to transmit the coronavirus because of being | N | 170 | 426 | 100 | 18 | 1 |
|  | Allergic | 74 (43.5%) | 162 (38.0%) | 46 (46.0%) | 5 (27.8%) | 1 (100.0%) |
|  | Under desensitization treatment | 5 (2.9%) | 5 (1.2%) | 1 (1.0%) | 1 (5.6%) | 0 |
|  | Allergic and under desensitization treatment | 10 (5.9%) | 40 (9.4%) | 4 (4.0%) | 2 (11.1%) | 0 |
|  | No reason specified | 81 (47.6%) | 219 (51.4%) | 49 (49.0%) | 10 (55.6%) | 0 |
| If allergic, type of allergy | N (mv) | 74 (0) | 159 (3) | 46 (0) | 5 (0) | 1 (0) |
|  | Allergic asthma | 29 (39.2%) | 71 (44.7%) | 22 (47.8%) | 2 (40.0%) | 0 |
|  | Allergic rhinoconjunctivitis | 45 (60.8%) | 88 (55.3%) | 24 (52.2%) | 3 (60.0%) | 1 (100.0%) |
| Feeling at risk to present severe symptoms of coronavirus | N (mv) | 901 (2) | 1957 (7) | 593 (5) | 138 (7) | 22 (0) |
|  | No | 706 (78.4%) | 1,500 (76.6%) | 448 (75.5%) | 109 (79.0%) | 18 (81.8%) |
|  | Yes | 195 (21.6%) | 457 (23.4%) | 145 (24.5%) | 29 (21.0%) | 4 (18.2%) |
| Feeling at risk to present severe symptoms of coronavirus because of being | N | 195 | 457 | 145 | 29 | 4 |
|  | Allergic | 156 (80.0%) | 339 (74.2%) | 107 (73.8%) | 21 (72.4%) | 1 (25.0%) |
|  | Under desensitization treatment | 0 | 1 (0.2%) | 1 (0.7%) | 0 | 0 |
|  | Allergic and under desensitization treatment | 23 (11.8%) | 56 (12.3%) | 13 (9.0%) | 1 (3.4%) | 2 (50.0%) |
|  | No reason specified | 16 (8.2%) | 61 (13.3%) | 24 (16.6%) | 7 (24.1%) | 1 (25.0%) |
| If allergic, type of allergy | N (mv) | 156 (0) | 337 (2) | 107 (0) | 21 (0) | 1 (0) |
|  | Allergic rhinoconjunctivitis | 28 (17.9%) | 72 (21.4%) | 25 (23.4%) | 8 (38.1%) | 1 (100.0%) |
| Data are n (%); mv = missing values  Due to rounding, total percentages may differ from 100.0% | | | | | | |

**Table S9.** AIT treatment according to type of comorbidities by SOC*.

|  | | **Cardiac disorders N = 76 (25.2%)** | **Metabolism and nutrition disorders N = 190 (62.9%)** | **Respiratory, thoracic and mediastinal disorders N = 115 (38.1%)** | **Neoplasms benign, malignant and unspecified N = 15 (5%)** |  |
| --- | --- | --- | --- | --- | --- | --- |
| Type of AIT treatment | N (mv) | 76 (0) | 188 (2) | 114 (1) | 15 (0) |  |
|  | Liquid | 67 (88.2%) | 155 (82.4%) | 87 (76.3%) | 12 (80.0%) |  |
|  | Liquid and tablet | 9 (11.8%) | 33 (17.6%) | 27 (23.7%) | 3 (20.0%) |  |
| Initiation of AIT before pandemic | N (mv) | 76 (0) | 189 (1) | 115 (0) | 15 (0) |  |
|  | No | 5 (6.6%) | 10 (5.3%) | 7 (6.1%) | 0 |  |
|  | Yes | 71 (93.4%) | 179 (94.7%) | 108 (93.9%) | 15 (100.0%) |  |
| If initiation of AIT before pandemic, modification of treatment during pandemic | N (mv) | 71 (0) | 179 (0) | 107 (1) | 15 (0) |  |
|  | Unchanged | 54 (76.1%) | 127 (70.9%) | 77 (72.0%) | 11 (73.3%) |  |
|  | Changed | 0 | 2 (1.1%) | 1 (0.9%) | 0 |  |
|  | Temporary interruption | 4 (5.6%) | 12 (6.7%) | 8 (7.5%) | 2 (13.3%) |  |
|  | Permanently discontinued | 13 (18.3%) | 38 (21.2%) | 21 (19.6%) | 2 (13.3%) |  |
| If treatment changed, type of modification | N | 0 | 2 | 1 | 0 |  |
|  | Initiation dose | 0 | 0 | 1 (100.0%) | 0 |  |
|  | Maintenance dose | 0 | 2 (100.0%) | 0 | 0 |  |
|  | Duration of treatment | 0 | 0 | 0 | 0 |  |
| If treatment changed, temporary interrupted or permanently discontinued, reappearance of allergic symptoms | N | 17 | 52 | 30 | 4 |  |
|  | No | 7 (41.2%) | 16 (30.8%) | 8 (26.7%) | 2 (50.0%) |  |
|  | Yes | 10 (58.8%) | 36 (69.2%) | 22 (73.3%) | 2 (50.0%) |  |
| If treatment changed, temporary interrupted or permanently discontinued, caused by COVID-19 infection or positive diagnosis | N | 17 | 52 | 30 | 4 |  |
|  | No | 16 (94.1%) | 50 (96.2%) | 28 (93.3%) | 4 (100.0%) |  |
|  | Yes | 1 (5.9%) | 2 (3.8%) | 2 (6.7%) | 0 |  |
| If no initiation of AIT before pandemic, postponement of treatment because of pandemic | N (mv) | 5 (0) | 10 (0) | 6 (1) | 0 (0) |  |
|  | No | 0 | 0 | 0 | 0 |  |
|  | Yes | 5 (100.0%) | 10 (100.0%) | 6 (100.0%) | 0 |  |
| Corticosteroid-based treatment uptake | N (mv) | 75 (1) | 189 (1) | 114 (1) | 15 (0) |  |
|  | No | 47 (62.7%) | 97 (51.3%) | 42 (36.8%) | 9 (60.0%) |  |
|  | Yes | 28 (37.3%) | 92 (48.7%) | 72 (63.2%) | 6 (40.0%) |  |
| Data are n (%); mv = missing values  Due to rounding, total percentages may differ from 100.0%  *One patient could suffer from more than one comorbidity | | | | | | |

**Table S10.** COVID-19 risk perception according to type of comorbidities by SOC*.

|  | | **Cardiac disorders N = 76 (25.2%)** | **Metabolism and nutrition disorders N = 190 (62.9%)** | **Respiratory, thoracic and mediastinal disorders N = 115 (38.1%)** | **Neoplasms benign, malignant and unspecified N = 15 (5%)** |  |
| --- | --- | --- | --- | --- | --- | --- |
| Feeling anxiety | N | 76 | 190 | 115 | 15 |  |
|  | Extremely | 5 (6.6%) | 10 (5.3%) | 15 (13.0%) | 1 (6.7%) |  |
|  | Very | 5 (6.6%) | 25 (13.2%) | 23 (20.0%) | 2 (13.3%) |  |
|  | Moderately | 17 (22.4%) | 46 (24.2%) | 20 (17.4%) | 3 (20.0%) |  |
|  | A little | 24 (31.6%) | 40 (21.1%) | 24 (20.9%) | 4 (26.7%) |  |
|  | Not at all | 25 (32.9%) | 69 (36.3%) | 33 (28.7%) | 5 (33.3%) |  |
| Feeling anxiety because of being | N | 51 | 121 | 82 | 10 |  |
|  | Allergic | 24 (47.1%) | 70 (57.9%) | 58 (70.7%) | 4 (40.0%) |  |
|  | Under desensitization treatment | 1 (2.0%) | 2 (1.7%) | 0 | 0 |  |
|  | Allergic and under desensitization treatment | 9 (17.6%) | 12 (9.9%) | 12 (14.6%) | 0 |  |
|  | No reason specified | 17 (33.3%) | 37 (30.6%) | 12 (14.6%) | 6 (60.0%) |  |
| If allergic, type of allergy | N (mv) | 23 (1) | 70 (0) | 58 (0) | 4 (0) |  |
|  | Allergic asthma | 17 (73.9%) | 51 (72.9%) | 49 (84.5%) | 3 (75.0%) |  |
|  | Allergic rhinoconjunctivitis | 6 (26.1%) | 19 (27.1%) | 9 (15.5%) | 1 (25.0%) |  |
| Feeling vulnerability | N (mv) | 75 (1) | 190 (0) | 114 (1) | 15 (0) |  |
|  | Extremely | 5 (6.7%) | 15 (7.9%) | 16 (14.0%) | 0 |  |
|  | Very | 9 (12.0%) | 29 (15.3%) | 32 (28.1%) | 3 (20.0%) |  |
|  | Moderately | 20 (26.7%) | 56 (29.5%) | 20 (17.5%) | 3 (20.0%) |  |
|  | A little | 20 (26.7%) | 34 (17.9%) | 24 (21.1%) | 2 (13.3%) |  |
|  | Not at all | 21 (28.0%) | 56 (29.5%) | 22 (19.3%) | 7 (46.7%) |  |
| Feeling vulnerability because of being | N | 54 | 134 | 92 | 8 |  |
|  | Allergic | 24 (44.4%) | 77 (57.5%) | 64 (69.6%) | 4 (50.0%) |  |
|  | Under desensitization treatment | 1 (1.9%) | 1 (0.7%) | 0 | 0 |  |
|  | Allergic and under desensitization treatment | 8 (14.8%) | 18 (13.4%) | 16 (17.4%) | 0 |  |
|  | No reason specified | 21 (38.9%) | 38 (28.4%) | 12 (13.0%) | 4 (50.0%) |  |
| If allergic, type of allergy | N (mv) | 24 (0) | 77 (0) | 64 (0) | 3 (1) |  |
|  | Allergic asthma | 22 (91.7%) | 58 (75.3%) | 56 (87.5%) | 3 (100.0%) |  |
|  | Allergic rhinoconjunctivitis | 2 (8.3%) | 19 (24.7%) | 8 (12.5%) | 0 |  |
| Feeling at risk to transmit the coronavirus | N | 76 | 190 | 115 | 15 |  |
|  | No | 57 (75.0%) | 128 (67.4%) | 84 (73.0%) | 12 (80.0%) |  |
|  | Yes | 19 (25.0%) | 62 (32.6%) | 31 (27.0%) | 3 (20.0%) |  |
| Feeling at risk to transmit the coronavirus because of being | N | 19 | 62 | 31 | 3 |  |
|  | Allergic | 4 (21.1%) | 24 (38.7%) | 19 (61.3%) | 0 |  |
|  | Under desensitization treatment | 0 | 1 (1.6%) | 0 | 0 |  |
|  | Allergic and under desensitization treatment | 4 (21.1%) | 6 (9.7%) | 6 (19.4%) | 0 |  |
|  | No reason specified | 11 (57.9%) | 31 (50.0%) | 6 (19.4%) | 3 (100.0%) |  |
| If allergic, type of allergy | N | 4 | 24 | 19 | 0 |  |
|  | Allergic asthma | 2 (50.0%) | 12 (50.0%) | 13 (68.4%) | 0 |  |
|  | Allergic rhinoconjunctivitis | 2 (50.0%) | 12 (50.0%) | 6 (31.6%) | 0 |  |
| Feeling at risk to present severe symptoms of coronavirus | N (mv) | 75 (1) | 190 (0) | 114 (1) | 15 (0) |  |
|  | No | 32 (42.7%) | 79 (41.6%) | 28 (24.6%) | 8 (53.3%) |  |
|  | Yes | 43 (57.3%) | 111 (58.4%) | 86 (75.4%) | 7 (46.7%) |  |
| Feeling at risk to present severe symptoms of coronavirus because of being | N | 43 | 111 | 86 | 7 |  |
|  | Allergic | 26 (60.5%) | 70 (63.1%) | 63 (73.3%) | 4 (57.1%) |  |
|  | Under desensitization treatment | 1 (2.3%) | 0 | 0 | 0 |  |
|  | Allergic and under desensitization treatment | 4 (9.3%) | 11 (9.9%) | 12 (14.0%) | 0 |  |
|  | No reason specified | 12 (27.9%) | 30 (27.0%) | 11 (12.8%) | 3 (42.9%) |  |
| If allergic, type of allergy | N | 26 | 70 | 63 | 4 |  |
|  | Allergic asthma | 23 (88.5%) | 54 (77.1%) | 50 (79.4%) | 3 (75.0%) |  |
|  | Allergic rhinoconjunctivitis | 3 (11.5%) | 16 (22.9%) | 13 (20.6%) | 1 (25.0%) |  |
| Data are n (%); mv = Missing values  Due to rounding, total percentages may differ from 100.0%  *One patient could suffer from more than one comorbidity | | | | | | |

**Table S11.** AIT treatment according to seniority of allergic respiratory disease.

|  | | **[0* - 4] years N = 403 (11.0%)** | **[5 - 14] years N = 1,332 (36.2%)** | **≥ 15 years N = 1,942 (52.8%)** | |
| --- | --- | --- | --- | --- | --- |
| Type of AIT treatment | N (mv) | 401 (2) | 1,327 (5) | 1,933 (9) | |
|  | Liquid | 319 (79.6%) | 1,049 (79.1%) | 1,516 (78.4%) | |
|  | Liquid and tablet | 82 (20.4%) | 278 (20.9%) | 417 (21.6%) | |
| Initiation of AIT before pandemic | N (mv) | 401 (2) | 1,329 (3) | 1,933 (9) | |
|  | No | 17 (4.2%) | 40 (3.0%) | 55 (2.8%) | |
|  | Yes | 384 (95.8%) | 1,289 (97.0%) | 1,878 (97.2%) | |
| If initiation of AIT before pandemic, modification of treatment during pandemic | N (mv) | 383 (1) | 1,285 (4) | 1,876 (2) | |
|  | Unchanged | 305 (79.6%) | 994 (77.4%) | 1,470 (78.4%) | |
|  | Changed | 6 (1.6%) | 20 (1.6%) | 42 (2.2%) | |
|  | Temporary interruption | 25 (6.5%) | 89 (6.9%) | 116 (6.2%) | |
|  | Permanently discontinued | 47 (12.3%) | 182 (14.2%) | 248 (13.2%) | |
| If treatment changed, type of modification | N | 6 | 20 | 42 | |
|  | Initiation dose | 2 (33.3%) | 8 (40.0%) | 8 (19.0%) | |
|  | Maintenance dose | 4 (66.7%) | 10 (50.0%) | 29 (69.0%) | |
|  | Duration of treatment | 0 | 2 (10.0%) | 5 (11.9%) | |
| If treatment changed, temporary interrupted or permanently discontinued, reappearance of allergic symptoms | N (mv) | 78 (0) | 290 (1) | 405 (1) | |
|  | No | 29 (37.2%) | 102 (35.2%) | 156 (38.5%) | |
|  | Yes | 49 (62.8%) | 188 (64.8%) | 249 (61.5%) | |
| If treatment changed, temporary interrupted or permanently discontinued, caused by COVID-19 infection or positive diagnosis | N | 78 | 291 | 406 | |
|  | No | 76 (97.4%) | 278 (95.5%) | 388 (95.6%) | |
|  | Yes | 2 (2.6%) | 13 (4.5%) | 18 (4.4%) | |
| If no initiation of AIT before pandemic, postponement of treatment because of pandemic | N (mv) | 13 (4) | 31 (9) | 46 (9) | |
|  | No | 0 | 0 | 0 | |
|  | Yes | 13 (100.0%) | 31 (100.0%) | 46 (100.0%) | |
| Corticosteroid-based treatment uptake | N (mv) | 396 (7) | 1,323 (9) | 1,924 (18) | |
|  | No | 285 (72.0%) | 906 (68.5%) | 1,138 (59.1%) | |
|  | Yes | 111 (28.0%) | 417 (31.5%) | 786 (40.9%) | |
| Data are n (%); mv = missing values  *0 means <1 year  **Over 36.0% of the respondents declared they have corticosteroids treatment, which is considered as a high proportion, a sensitivity analysis of patients with corticosteroids by oral and inhaled routes as compared to the other patients was performed. | | | | |  |

**Table S12.** COVID-19 risk perception according to seniority of allergic respiratory disease.

|  | | **[0* - 4] years N = 403 (11.0%)** | **[5 - 14] years N = 1,332 (36.2%)** | **≥ 15 years N = 1,942 (52.8%)** | |
| --- | --- | --- | --- | --- | --- |
| Feeling anxiety | N (mv) | 403 (0) | 1,331 (1) | 1,938 (4) | |
|  | Extremely | 13 (3.2%) | 32 (2.4%) | 63 (3.3%) | |
|  | Very | 25 (6.2%) | 77 (5.8%) | 145 (7.5%) | |
|  | Moderately | 62 (15.4%) | 219 (16.5%) | 353 (18.2%) | |
|  | A little | 61 (15.1%) | 218 (16.4%) | 377 (19.5%) | |
|  | Not at all | 242 (60.0%) | 785 (59.0%) | 1,000 (51.6%) | |
| Feeling anxiety because of being | N | 161 | 546 | 938 | |
|  | Allergic | 80 (49.7%) | 249 (45.6%) | 482 (51.4%) | |
|  | Under desensitization treatment | 13 (8.1%) | 31 (5.7%) | 21 (2.2%) | |
|  | Allergic and under desensitization treatment | 25 (15.5%) | 68 (12.5%) | 120 (12.8%) | |
|  | No reason specified | 43 (26.7%) | 198 (36.3%) | 315 (33.6%) | |
| If allergic, type of allergy | N (mv) | 78 (2) | 244 (5) | 473 (9) | |
|  | Allergic asthma | 50 (64.1%) | 116 (47.5%) | 293 (61.9%) | |
|  | Allergic rhinoconjunctivitis | 28 (35.9%) | 128 (52.5%) | 180 (38.1%) | |
| Feeling vulnerability | N (mv) | 402 (1) | 1,328 (4) | 1,936 (6) | |
|  | Extremely | 11 (2.7%) | 11 (0.8%) | 45 (2.3%) | |
|  | Very | 20 (5.0%) | 65 (4.9%) | 158 (8.2%) | |
|  | Moderately | 66 (16.4%) | 217 (16.3%) | 367 (19.0%) | |
|  | A little | 77 (19.2%) | 272 (20.5%) | 413 (21.3%) | |
|  | Not at all | 228 (56.7%) | 763 (57.5%) | 953 (49.2%) | |
| Feeling vulnerability because of being | N | 174 | 565 | 983 | |
|  | Allergic | 92 (52.9%) | 337 (59.6%) | 596 (60.6%) | |
|  | Under desensitization treatment | 10 (5.7%) | 21 (3.7%) | 22 (2.2%) | |
|  | Allergic and under desensitization treatment | 29 (16.7%) | 58 (10.3%) | 114 (11.6%) | |
|  | No reason specified | 43 (24.7%) | 149 (26.4%) | 251 (25.5%) | |
| If allergic, type of allergy | N (mv) | 92 (0) | 333 (4) | 590 (6) | |
|  | Allergic asthma | 58 (63.0%) | 189 (56.8%) | 392 (66.4%) | |
|  | Allergic rhinoconjunctivitis | 34 (37.0%) | 144 (43.2%) | 198 (33.6%) | |
| Feeling at risk to transmit the coronavirus | N (mv) | 402 (1) | 1,327 (5) | 1,937 (5) | |
|  | No | 329 (81.8%) | 1,086 (81.8%) | 1,530 (79.0%) | |
|  | Yes | 73 (18.2%) | 241 (18.2%) | 407 (21.0%) | |
| Feeling at risk to transmit the coronavirus because of being | N | 73 | 241 | 407 | |
|  | Allergic | 29 (39.7%) | 92 (38.2%) | 169 (41.5%) | |
|  | Under desensitization treatment | 4 (5.5%) | 3 (1.2%) | 4 (1.0%) | |
|  | Allergic and under desensitization treatment | 7 (9.6%) | 15 (6.2%) | 34 (8.4%) | |
|  | No reason specified | 33 (45.2%) | 131 (54.4%) | 200 (49.1%) | |
| If allergic, type of allergy | N (mv) | 28 (1) | 92 (0) | 166 (3) | |
|  | Allergic asthma | 14 (50.0%) | 32 (34.8%) | 79 (47.6%) | |
|  | Allergic rhinoconjunctivitis | 14 (50.0%) | 60 (65.2%) | 87 (52.4%) | |
| Feeling at risk to present severe symptoms of coronavirus | N (mv) | 400 (3) | 1328 (4) | 1929 (13) | |
|  | No | 322 (80.5%) | 1,089 (82.0%) | 1,409 (73.0%) | |
|  | Yes | 78 (19.5%) | 239 (18.0%) | 520 (27.0%) | |
| Feeling at risk to present severe symptoms of coronavirus because of being | N | 78 | 239 | 520 | |
|  | Allergic | 54 (69.2%) | 179 (74.9%) | 397 (76.3%) | |
|  | Under desensitization treatment | 0 | 1 (0.4%) | 1 (0.2%) | |
|  | Allergic and under desensitization treatment | 17 (21.8%) | 22 (9.2%) | 57 (11.0%) | |
|  | No reason specified | 7 (9.0%) | 37 (15.5%) | 65 (12.5%) | |
| If allergic, type of allergy | N (mv) | 53 (1) | 178 (1) | 396 (1) | |
|  | Allergic asthma | 44 (83.0%) | 128 (71.9%) | 322 (81.3%) | |
|  | Allergic rhinoconjunctivitis | 9 (17.0%) | 50 (28.1%) | 74 (18.7%) | |
| Data are n (%); mv = missing values  *0 means <1 year | | | | |  |

**Table S13.** AIT treatment according to allergy status.

|  | | **Mono-allergic N = 1,015 (27.3%)** | **Poly-allergic N = 2,701 (72.7%)** |
| --- | --- | --- | --- |
| Type of AIT treatment | N (mv) | 1,011 (4) | 2,688 (13) |
|  | Liquid | 866 (85.7%) | 2,048 (76.2%) |
|  | Liquid and tablet | 145 (14.3%) | 640 (23.8%) |
| Initiation of AIT before pandemic | N (mv) | 1,011 (4) | 2,691 (10) |
|  | No | 28 (2.8%) | 86 (3.2%) |
|  | Yes | 983 (97.2%) | 2,605 (96.8%) |
| If initiation of AIT before pandemic, modification of treatment during pandemic | N (mv) | 980 (3) | 2,601 (4) |
|  | Unchanged | 791 (80.7%) | 2,008 (77.2%) |
|  | Changed | 12 (1.2%) | 56 (2.2%) |
|  | Temporary interruption | 57 (5.8%) | 178 (6.8%) |
|  | Permanently discontinued | 120 (12.2%) | 359 (13.8%) |
| If treatment changed, type of modification | N | 12 | 56 |
|  | Initiation dose | 1 (8.3%) | 17 (30.4%) |
|  | Maintenance dose | 9 (75.0%) | 34 (60.7%) |
|  | Duration of treatment | 2 (16.7%) | 5 (8.9%) |
| If treatment changed, temporary interrupted or permanently discontinued, reappearance of allergic symptoms | N (mv) | 188 (1) | 592 (1) |
|  | No | 89 (47.3%) | 201 (34.0%) |
|  | Yes | 99 (52.7%) | 391 (66.0%) |
| If treatment changed, temporary interrupted or permanently discontinued, caused by COVID-19 infection or positive diagnosis | N | 189 | 593 |
|  | No | 185 (97.9%) | 564 (95.1%) |
|  | Yes | 4 (2.1%) | 29 (4.9%) |
| If no initiation of AIT before pandemic, postponement of treatment because of pandemic | N (mv) | 23 (5) | 68 (18) |
|  | No | 0 | 0 |
|  | Yes | 23 (100.0%) | 68 (100.0%) |
| Corticosteroid-based treatment uptake | N (mv) | 1,005 (10) | 2,677 (24) |
|  | No | 725 (72.1%) | 1,632 (61.0%) |
|  | Yes | 280 (27.9%) | 1,045 (39.0%) |
| Data are n (%); mv = missing values | | | |

**Table S14.** COVID-19 risk perception according to allergy status.

|  | | **Mono-allergic N = 1,015 (27.3%)** | **Poly-allergic N = 2,701 (72.7%)** | |
| --- | --- | --- | --- | --- |
| Feeling anxiety | N (mv) | 1,013 (2) | 2,697 (4) | |
|  | Extremely | 22 (2.2%) | 86 (3.2%) | |
|  | Very | 47 (4.6%) | 202 (7.5%) | |
|  | Moderately | 144 (14.2%) | 498 (18.5%) | |
|  | A little | 164 (16.2%) | 499 (18.5%) | |
|  | Not at all | 636 (62.8%) | 1,412 (52.4%) | |
| Feeling anxiety because of being | N | 377 | 1,285 | |
|  | Allergic | 169 (44.8%) | 648 (50.4%) | |
|  | Under desensitization treatment | 16 (4.2%) | 50 (3.9%) | |
|  | Allergic and under desensitization treatment | 45 (11.9%) | 169 (13.2%) | |
|  | No reason specified | 147 (39.0%) | 418 (32.5%) | |
| If allergic, type of allergy | N (mv) | 167 (2) | 634 (14) | |
|  | Allergic asthma | 84 (50.3%) | 377 (59.5%) | |
|  | Allergic rhinoconjunctivitis | 83 (49.7%) | 257 (40.5%) | |
| Feeling vulnerability | N (mv) | 1,011 (4) | 2,692 (9) | |
|  | Extremely | 18 (1.8%) | 51 (1.9%) | |
|  | Very | 40 (4.0%) | 205 (7.6%) | |
|  | Moderately | 147 (14.5%) | 508 (18.9%) | |
|  | A little | 191 (18.9%) | 577 (21.4%) | |
|  | Not at all | 615 (60.8%) | 1,351 (50.2%) | |
| Feeling vulnerability because of being | N | 396 | 1,341 | |
|  | Allergic | 221 (55.8%) | 809 (60.3%) | |
|  | Under desensitization treatment | 16 (4.0%) | 39 (2.9%) | |
|  | Allergic and under desensitization treatment | 48 (12.1%) | 154 (11.5%) | |
|  | No reason specified | 111 (28.0%) | 339 (25.3%) | |
| If allergic, type of allergy | N (mv) | 218 (3) | 802 (7) | |
|  | Allergic asthma | 115 (52.8%) | 529 (66.0%) | |
|  | Allergic rhinoconjunctivitis | 103 (47.2%) | 273 (34.0%) | |
| Feeling at risk to transmit the coronavirus | N (mv) | 1,010 (5) | 2,693 (8) | |
|  | No | 846 (83.8%) | 2,131 (79.1%) | |
|  | Yes | 164 (16.2%) | 562 (20.9%) | |
| Feeling at risk to transmit the coronavirus because of being | N | 164 | 562 | |
|  | Allergic | 72 (43.9%) | 221 (39.3%) | |
|  | Under desensitization treatment | 4 (2.4%) | 8 (1.4%) | |
|  | Allergic and under desensitization treatment | 10 (6.1%) | 46 (8.2%) | |
|  | No reason specified | 78 (47.6%) | 287 (51.1%) | |
| If allergic, type of allergy | N (mv) | 71 (1) | 218 (3) | |
|  | Allergic asthma | 26 (36.6%) | 101 (46.3%) | |
|  | Allergic rhinoconjunctivitis | 45 (63.4%) | 117 (53.7%) | |
| Feeling at risk to present severe symptoms of coronavirus | N (mv) | 1007 (8) | 2688 (13) | |
|  | No | 833 (82.7%) | 2,017 (75.0%) | |
|  | Yes | 174 (17.3%) | 671 (25.0%) | |
| Feeling at risk to present severe symptoms of coronavirus because of being | N | 174 | 671 | |
|  | Allergic | 127 (73.0%) | 509 (75.9%) | |
|  | Under desensitization treatment | 0 (0.0%) | 2 (0.3%) | |
|  | Allergic and under desensitization treatment | 16 (9.2%) | 80 (11.9%) | |
|  | No reason specified | 31 (17.8%) | 80 (11.9%) | |
| If allergic, type of allergy | N (mv) | 127 (0) | 506 (3) | |
|  | Allergic asthma | 88 (69.3%) | 409 (80.8%) | |
|  | Allergic rhinoconjunctivitis | 39 (30.7%) | 97 (19.2%) | |
| Data are n (%); mv = missing values | | | |  |

**Table S15.** COVID-19 risk perception according to allergic asthma status.

|  | | **Asthmatic N = 1,451 (39.0%)** | **Non-asthmatic N = 2,272 (61.0%)** |
| --- | --- | --- | --- |
| Feeling anxiety | N (mv) | 1,449 (2) | 2,267 (5) |
|  | Extremely | 55 (3.8%) | 53 (2.3%) |
|  | Very | 130 (9.0%) | 119 (5.2%) |
|  | Moderately | 302 (20.8%) | 341 (15.0%) |
|  | A little | 285 (19.7%) | 379 (16.7%) |
|  | Not at all | 677 (46.7%) | 1,375 (60.7%) |
| Feeling anxiety because of being | N | 772 | 892 |
|  | Allergic | 477 (61.8%) | 341 (38.2%) |
|  | Under desensitization treatment | 23 (3.0%) | 43 (4.8%) |
|  | Allergic and under desensitization treatment | 109 (14.1%) | 105 (11.8%) |
|  | No reason specified | 163 (21.1%) | 403 (45.2%) |
| If allergic, type of allergy | N (mv) | 472 (5) | 330 (11) |
|  | Allergic asthma | 430 (91.1%) | 32 (9.7%) |
|  | Allergic rhinoconjunctivitis | 42 (8.9%) | 298 (90.3%) |
| Feeling vulnerability | N (mv) | 1,445 (6) | 2,264 (8) |
|  | Extremely | 49 (3.4%) | 20 (0.9%) |
|  | Very | 175 (12.1%) | 70 (3.1%) |
|  | Moderately | 333 (23.0%) | 323 (14.3%) |
|  | A little | 338 (23.4%) | 432 (19.1%) |
|  | Not at all | 550 (38.1%) | 1,419 (62.7%) |
| Feeling vulnerability because of being | N | 895 | 845 |
|  | Allergic | 650 (72.6%) | 381 (45.1%) |
|  | Under desensitization treatment | 17 (1.9%) | 38 (4.5%) |
|  | Allergic and under desensitization treatment | 104 (11.6%) | 98 (11.6%) |
|  | No reason specified | 124 (13.9%) | 328 (38.8%) |
| If allergic, type of allergy | N (mv) | 649 (1) | 372 (9) |
|  | Allergic asthma | 596 (91.8%) | 49 (13.2%) |
|  | Allergic rhinoconjunctivitis | 53 (8.2%) | 323 (86.8%) |
| Feeling at risk to transmit the coronavirus | N (mv) | 1,448 (3) | 2,261 (11) |
|  | No | 1,144 (79.0%) | 1,837 (81.2%) |
|  | Yes | 304 (21.0%) | 424 (18.8%) |
| Feeling at risk to transmit the coronavirus because of being | N | 304 | 424 |
|  | Allergic | 149 (49.0%) | 144 (34.0%) |
|  | Under desensitization treatment | 3 (1.0%) | 9 (2.1%) |
|  | Allergic and under desensitization treatment | 29 (9.5%) | 27 (6.4%) |
|  | No reason specified | 123 (40.5%) | 244 (57.5%) |
| If allergic, type of allergy | N | 148 (1) | 141 (3) |
|  | Allergic asthma | 115 (77.7%) | 12 (8.5%) |
|  | Allergic rhinoconjunctivitis | 33 (22.3%) | 129 (91.5%) |
| Feeling at risk to present severe symptoms of coronavirus | N (mv) | 1,440 (11) | 2,261 (11) |
|  | No | 850 (59.0%) | 2,005 (88.7%) |
|  | Yes | 590 (41.0%) | 256 (11.3%) |
| Feeling at risk to present severe symptoms of coronavirus because of being | N | 590 | 256 |
|  | Allergic | 474 (80.3%) | 163 (63.7%) |
|  | Under desensitization treatment | 1 (0.2%) | 1 (0.4%) |
|  | Allergic and under desensitization treatment | 72 (12.2%) | 24 (9.4%) |
|  | No reason specified | 43 (7.3%) | 68 (26.6%) |
| If allergic, type of allergy | N (mv) | 474 (0) | 160 (3) |
|  | Allergic asthma | 456 (96.2%) | 42 (26.3%) |
|  | Allergic rhinoconjunctivitis | 18 (3.8%) | 118 (73.8%) |
| Data are n (%); mv = missing values | | | |


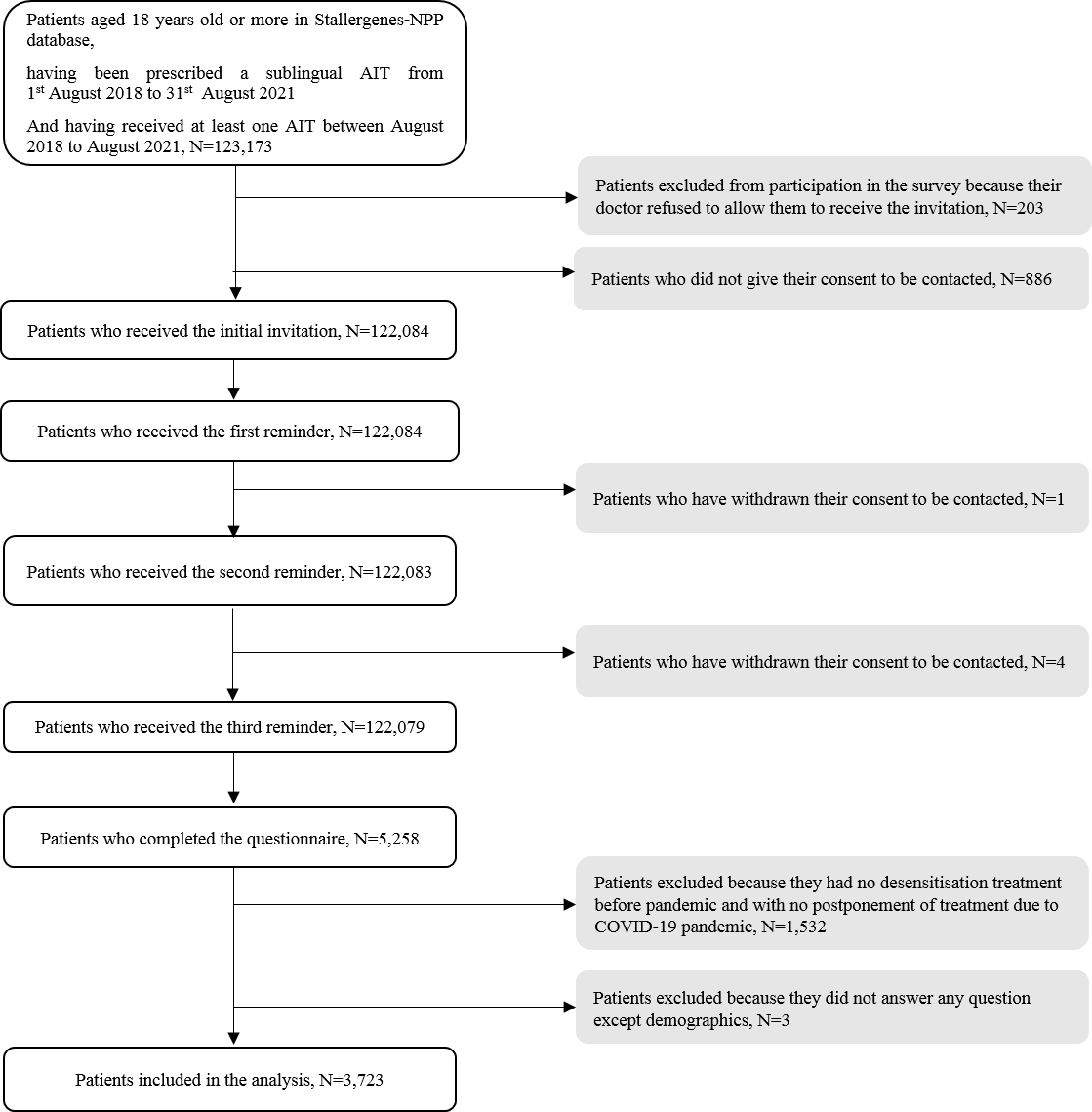


**Figure S1.** Patient disposition.
